# Recent time trends in incidence, outcome and premorbid treatment of atrial fibrillation-related stroke and other embolic vascular events: a population-based study

**DOI:** 10.1136/jnnp-2015-311947

**Published:** 2015-10-20

**Authors:** Gabriel S C Yiin, Dominic P J Howard, Nicola L M Paul, Linxin Li, Ziyah Mehta, Peter M Rothwell

**Affiliations:** Stroke Prevention Research Unit, Nuffield Department of Clinical Neurosciences, University of Oxford, Oxford, Oxfordshire, UK

## Abstract

**Background:**

Prevalence of atrial fibrillation (AF) is increasing, due partly to the ageing population. The Birmingham Atrial Fibrillation Treatment of the Aged (BAFTA) Trial, published in 2007, provided strong evidence of the effectiveness of warfarin at age≥80 years, but the impact on incidence of AF-related stroke and peripheral embolic vascular events is uncertain.

**Methods:**

We studied age-specific incidence and outcome of all AF-related incident strokes and systemic emboli from 2002 to 2012 in the Oxford Vascular Study.

**Results:**

Of 3096 acute cerebral or peripheral vascular events, 748 (24.2%) were AF-related. Of the 597 disabling/fatal incident ischaemic strokes, 369 occurred at age ≥80 years, of which 124 (33.6%) were in non-anticoagulated patients with known prior AF. There was no reduction in incident AF-related events after 2007 at all ages (n=231 vs 211; adjusted RR=1.11, 0.91 to 1.36, p=0.29) or at age ≥80 (137 vs 135, RR=1.15, 0.94 to 1.40, p=0.17). Scope for improved prevention at older ages was considerable. Among 208 patients with incident AF-related events at age ≥80 and known prior AF, only 19 (9.1%) were anticoagulated. Of the 189 patients not anticoagulated, 166 (87.8%) had no major disability prior to the event and 167 (88·4%) had a high embolism risk score, of whom 139 (83.2%) were also at low risk of complications. Yet, 125/167 (74.9%) were dead or institutionalised after the event. Potentially preventable embolic events outnumbered warfarin-related intracerebral haemorrhages by about 15-fold (280 vs 19), rising to 50-fold (189 vs 4) at age ≥80 years.

**Conclusions:**

We found no reduction in incidence of AF-related vascular events since publication of the BAFTA trial. A third of all disabling/fatal strokes occur in non-anticoagulated patients with known prior AF.

## Introduction

It is estimated that 1.1 million people in the UK have atrial fibrillation (AF),^1^ with age-specific prevalence increasing from 0.5% at 50–59 years of age to 10% at ≥80 years.^2^ AF is one of the most common preventable causes of stroke, conferring a fivefold increased risk of stroke and accounting for about 12 500 strokes a year in the UK.^3^ AF-related ischaemic strokes also tend to be severe and to incur high mean costs,^4^ and non-cerebral systemic embolism secondary to AF is also a major burden.^5^

Anticoagulation with warfarin is highly effective in primary prevention of AF-related embolic events,^6^ ^7^ associated with reduced stroke severity,^8^ ^9^ and several new oral anticoagulants have been shown to have equivalent or greater net clinical benefit.^10^ Yet, irrespective of which drugs are used, the overall impact of anticoagulation on AF-related ischaemic events at the population level has probably been small due to widespread under-treatment,^11–13^ particularly in the elderly (see online supplementary table S1a–c). We showed that there has, in fact, been a three-fold increase in AF-related ischaemic stroke at age ≥80 years in Oxfordshire from 1981–1986 to 2002–2012.^14^ However, screening for AF and prevention of stroke in primary care were incentivised with the introduction in the UK of the Quality and Outcomes Framework (QOF) in 2006 (see online supplementary table S2), and good evidence that warfarin is more effective than aspirin in primary prevention in high-risk elderly patients with AF was provided in 2007 with the results of the Birmingham Atrial Fibrillation Treatment of the Aged (BAFTA) trial.^15^ What impact these developments had on the incidence of AF-related vascular events in high-risk older people is uncertain.^1^ We, therefore, determined changes in age and sex-specific incidence of AF-related vascular events in Oxfordshire, UK, before and after 2007, including changes in rates of known prior AF and premorbid treatment in relation to age, sex, risk scores and contraindications, premorbid disability and clinical outcome.

## Methods

### Study population

OXVASC is a population-based study of the incidence and outcome of all acute vascular events in a mixed urban/rural population of Oxfordshire, UK. Methods and definitions of events (see online supplementary S3) have been reported previously.^16^ ^17^ Briefly, the study population comprises 92 728 individuals registered with nine general practices (about 100 family doctors) that refer patients to the main Oxford Hospitals. Ascertainment of acute vascular events started in 1 April 2002 and is on-going. Case ascertainment uses multiple overlapping methods of hot and cold pursuit (see online supplementary S3) and has been shown to be near complete.^17^ For this paper, only incident transient ischaemic attacks (TIAs), strokes and peripheral embolic vascular events (PVEs) ascertained up to 31 March 2012 were included. OXVASC has local research ethics committee approval.

All patients gave informed consent to participate in OXVASC, or assent was gained from a relative. Patients were seen by study physicians as soon as possible after presentation (assessment details in online supplementary S3). ECG and not ambulatory cardiac monitoring was performed routinely at baseline as part of clinical care. Clinical study reports of all strokes were reviewed by the senior study neurologist and reports of all PVEs were reviewed by a vascular surgeon. We obtained additional premorbid baseline clinical characteristics, lipid profile, BP measurements and medications by interviewing patients and relatives, and by review of primary care and hospital records.

Stroke was defined as an event with appropriate symptoms lasting longer than 24 h.^16^ PVEs included all cases of presumed embolic acute limb ischaemia or acute visceral ischaemia (including aortic, renal, splenic, hepatic and intestinal). AF-related events were defined as those associated with paroxysmal, persistent or permanent AF^18^ (defined on the basis of an ECG showing either absent p waves or atrial flutter with an irregular ventricular response) documented before the event, at the time of assessment, or within 1 month after the event. Patients were subdivided according to whether AF had been documented prior to the acute event (‘known prior AF’), with confirmation from primary care or hospital records.

In all patients with known prior AF, we used premorbid clinical characteristics to calculate the CHADS_2_ score^19^ and CHA_2_DS_2_VASc score^20^ for risk of embolic ischaemic events, and the HAS-BLED score^21^ for the risk of bleeding on anticoagulation. In those patients not on anticoagulation at the time of the event, we reviewed their primary care and hospital records to identify any written explanation as to why anticoagulation was not used and identified any reasons for previous discontinuation. Aetiological subtype of stroke was classified with the TOAST criteria (Trial of Org 10172).^22^

All patients had face-to-face follow-up at 1, 6, 12, 24, 60 and 120 months after the event to assess outcomes. For patients who had moved out of the study area, telephone follow-up was performed. Follow-up was conducted via a carer if the patient was unable to participate, for example, due to dementia. Institutionalisation was defined as living in a nursing home, residential home or community rehabilitation hospital. Major ischaemic stroke was defined as National Institutes of Health (NIH) stroke scale ≥5. Disabling/fatal stroke or PVE were defined as having a modified Rankin scale (mRS) score >2 at 6-months follow-up. Premorbid disability was also defined as mRS>2, with major disability defined as mRS>3 (ie, not independently mobile).

### Statistical analysis

Sex-specific rates (per 1000 population per year) of AF-related incident strokes and PVEs were calculated in 10-year age bands, with CIs estimated assuming a Poisson distribution. We used Poisson regression models to calculate the relative incidence of AF-related ischaemic event between time periods in OXVASC. We used χ^2^ or Fisher's Exact test to compare categorical variables and Student t test for continuous variables. Binary logistic regression was used to calculate the age-adjusted OR.

We performed statistical analysis and graphical presentation using SPSS software V.20.0, Microsoft Excel 2010 for Windows and SAS software V.9.2.

### Role of the funding source

The sponsor of the study had no role in the study design, data collection, data analysis, data interpretation, or writing of the report. All authors had access to the data and took responsibility for the decision to submit the manuscript.

## Results

Of 3096 acute cerebral or peripheral vascular events in OXVASC during 2002–2012, 748 (24.2%) were AF-related, including 601 incident events (383 ischaemic stroke, 122 TIA, 71 PVE and 25 intracerebral haemorrhage), of whom 442 (73.5%) had documented prior AF. Baseline characteristics are given in table 1.

**Table 1:** Baseline characteristics of patients with AF-related and non-AF-related incident ischaemic stroke

	Non-AF (n=865)	AF (n=383)	Premorbid AF (n=274)	Unadjusted p value*
Male sex (%)	457 (52.8)	171 (44·6)	126 (46·0)	0.008
Mean age (SD) (years)	73.0 (13.5)	80.0 (9·7)	80·4 (9·4)	<0.0001
Congestive cardiac failure	55 (6.4)	99 (25·8)	85 (31·0)	<0.0001
Hypertension	530 (61.3)	287 (74·9)	212 (77·4)	<0.0001
Diabetes	124 (14.3)	58 (15·1)	49 (17·9)	0.71
Previous TIA	98 (11.3)	61 (15·9)	46 (16·8)	0.025
Previous MI	91 (10.5)	72 (18·8)	59 (21·5)	0.0001
Angina	136 (15.7)	102 (26.6)	87 (31·8)	<0.0001
Current smoking	155 (17.9)	29 (7·6)	17 (6·2)	<0.0001
Hypercholesterolaemia†	276 (31.9)	118 (30·8)	88 (32·1)	0.70
Peripheral vascular disease	65 (7.5)	56 (14·6)	40 (14.6)	0.0001
Valvular heart disease	60 (6.9)	82 (21·4)	73 (26·6)	<0.0001
Venous thromboembolism	48 (5.5)	27 (7.0)	21 (7.7)	0.30
Antiplatelet agent(s)	293 (33.9)	202 (52·7)	167 (60·9)	<0.0001
Lipid lowering agent	214 (24.7)	102 (26·6)	82 (29·9)	0.48
Antihypertensive(s)	484 (56.0)	282 (73·1)	212 (77.4)	<0.0001
Anticoagulant	9 (1.0)	47 (12·3)	46 (16·8)	<0.0001

*Between AF and non-AF groups

†Defined as ≥6.0 mmol/L

AF, atrial fibrillation; MI, myocardial infarction; TIA, transient ischaemic attack.

**Figure 1:**
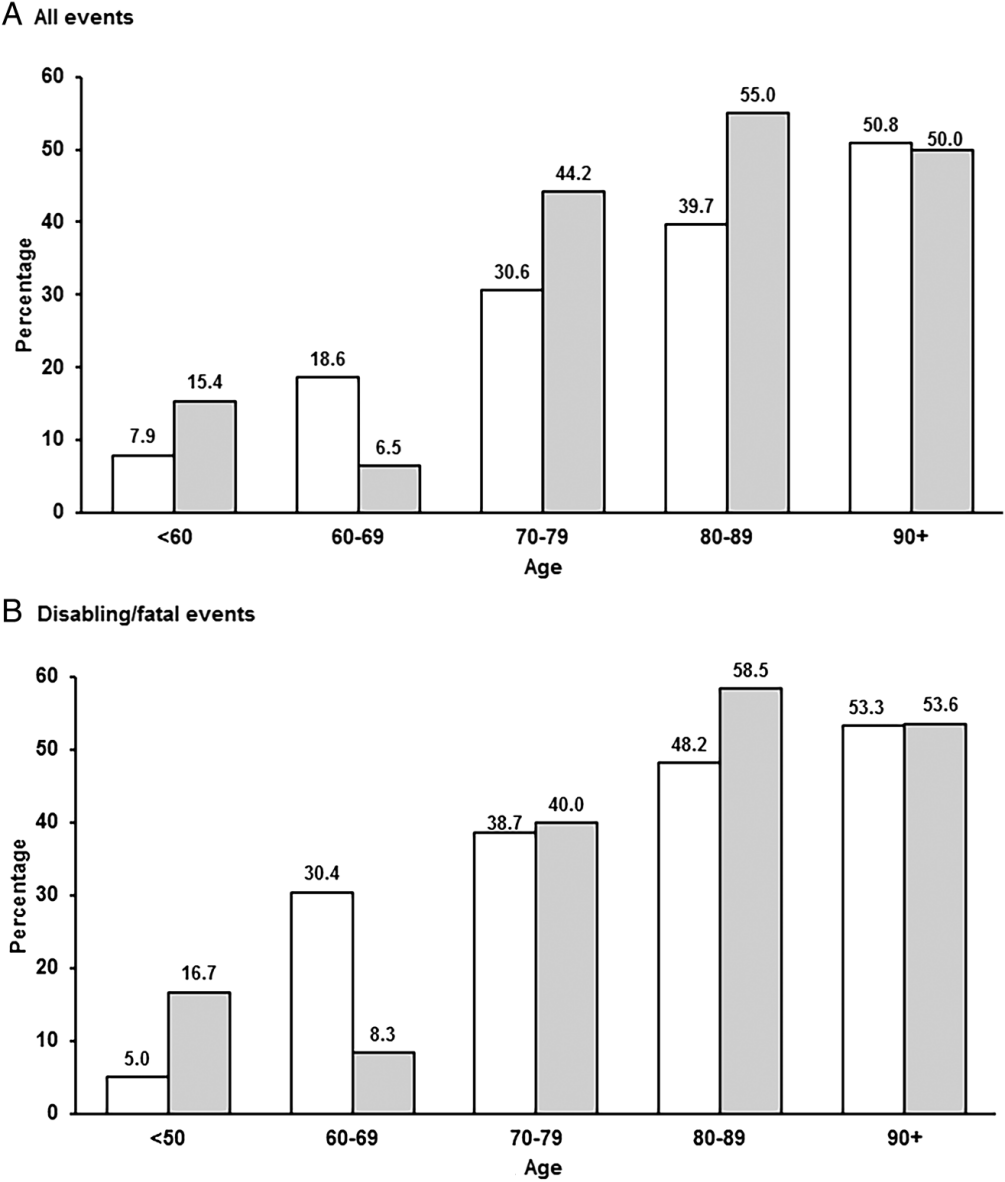
Proportion of incident ischaemic strokes (left bars) and peripheral embolic vascular events (right bars) related to all atrial fibrillation AF by age for all events (A) and disabling/fatal events (B).

**Figure 2:**
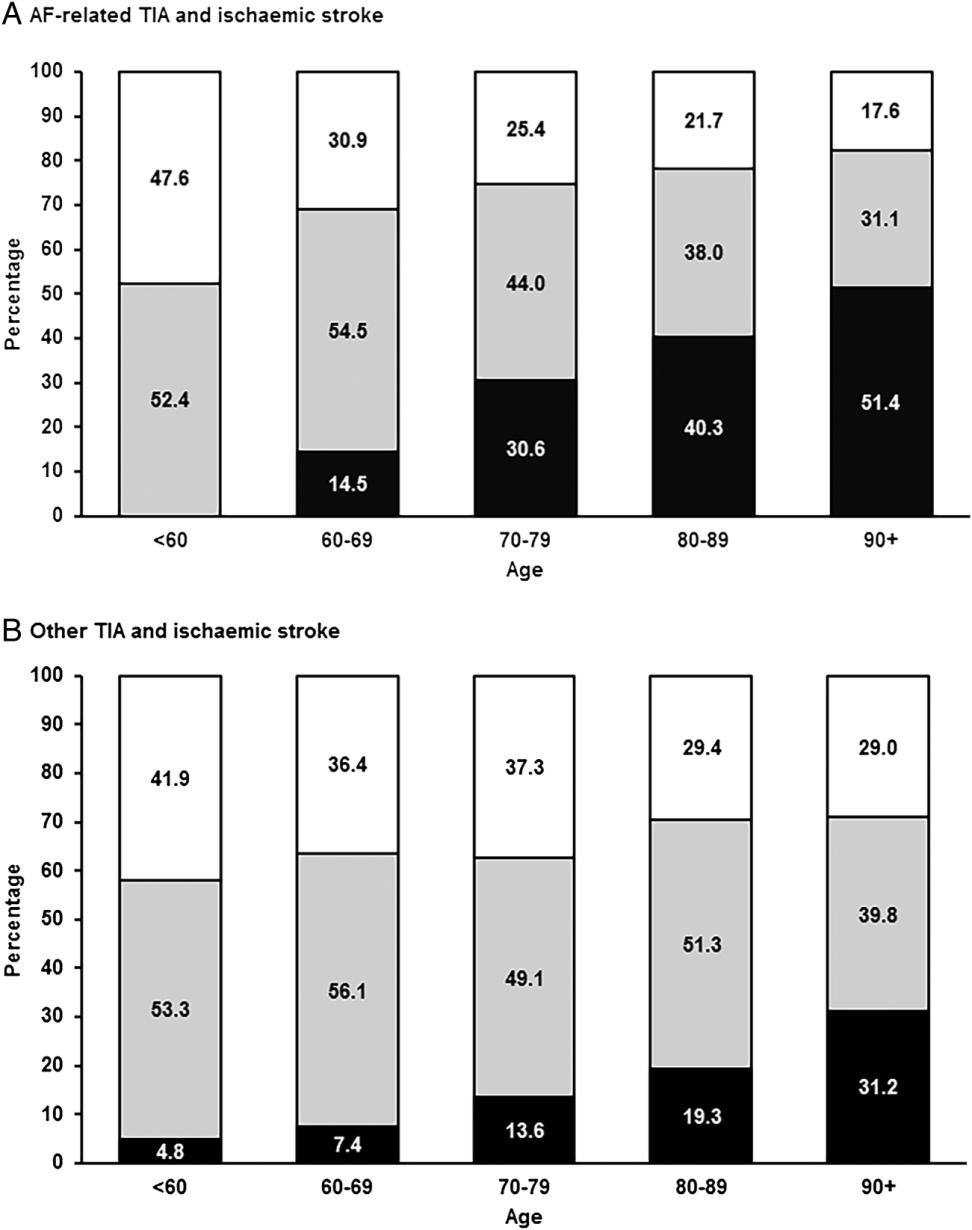
Distribution of incident atrial fibrillation (AF)-related (A; n=505) and non-AF-related (B; n=1337) transient ischaemic attacks (TIAs) and ischaemic strokes by age and severity; TIA (white), minor non-disabling ischaemic stroke (grey), major fatal/disabling ischaemic stroke (black).

Incidence rates of AF-related ischaemic stroke or PVE were similar in men and women, but increased steeply with age, with 272/454 (59.9%) events occurring at ≥80 years (table 2, online supplementary tables S4–5). Of 1425 patients with incident ischaemic stroke (1248) or PVE (177) in the study population, the proportion with AF-related events increased from 7.8% at age<60 years to 50.7% at age≥90 (figure 1). The severity of AF-related cerebral events also increased steeply with age (p<00001; figure 2), the proportion that were disabling or fatal ischaemic strokes reaching 51.4% by age ≥90 years.

**Table 2:** Age-specific rates per 1000 population per year of AF-related incident ischaemic stroke and peripheral embolic vascular event in OXVASC

Age (years)	Men	Rate per 1000 per year (95% CI)	Women	Rate per 1000 per year (95% CI)	Total	Rate per 1000 per year (95% CI)
<60	13/38 736	0.03 (0.02 to 0.06)	1/35 656	0.00 (0.00 to 0.02)	14/74 392	0.02 (0.01 to 0.03)
60–69	21/4308	0.49 (0.30 to 0.75)	21/4332	0.48 (0.30 to 0.74)	42/8640	0.49 (0.35 to 0.66)
70–79	65/2848	2.28 (1.76 to 2.91)	61/3187	1.91 (1.46 to 2.46)	126/6035	2.09 (1.74 to 2.49)
80–89	83/1207	6.88 (5.48 to 8.53)	111/1914	5.80 (4.77 to 6.98)	194/3121	6.22 (5.37 to 7.16)
≥90	16/147	10.90 (6.23 to 17.71)	62/393	15.79 (12.11 to 20.24)	78/540	14.46 (11.43 to 18.05)
Total	198/47 246	0.42 (0.36 to 0.48)	256/45 482	0.56 (0.50 to 0.64)	454/92 728	0.49 (0.45 to 0.54)
				Premorbid AF	336/92 728	0.36 (0.32 to 0.40)
				New AF	118/92 728	0.13 (0.11 to 0.15)

AF, atrial fibrillation.

There was no reduction in number of AF-related ischaemic strokes and PVEs between 2007–2012 and 2002–2007 (n=231 vs 223; identical incidence=0.49/1000/year, 95% CI 0.43 to 0.56; age/sex-adjusted RR 1.11, 0.91 to 1.36, p=0.29), with similar results for events at age ≥80 years (137 vs 135, RR=1.15, 0.94 to 1.40, p=0.17) and in paroxysmal AF-related events (74/223 vs 67/231, p=0.34). However, the rates of AF-related vascular events tended to be higher in 2010–2012 than in 2007–2009 (table 3) especially at age ≥80 years (8.03 vs 5.89/1000/year for any AF and 6.30 vs 5.28/1000/year for premorbid AF).

**Table 3:** Age-specific rates of AF-related incident ischaemic stroke and PVE at different time periods

Ischaemic stroke or PVE	All ages		Age <80		Age ≥80	
	Rate/1000/year	Number	Rate/1000/year	Number	Rate/1000/year	Number
Total AF						
2002–2012	0.49 (0.45–0.54)	454	0.20 (0.18–0.24)	182	7.43 (6.57–8.37)	272
2002–2007	0.49 (0.43–0.56)	223	0.20 (0.16–0.25)	88	7.98 (6.69–9.45)	135
2007–2012	0.49 (0.43–0.56)	231	0.21 (0.17–0.25)	94	6.96 (5.84–8.23)	137
2007–2009.5*	0.42 (0.34–0.51)	99	0.18 (0.13–0.25)	41	5.89 (4.47–7.62)	58
2009.5*–2012	0.56 (0.47–0.66)	132	0.23 (0.18–0.31)	53	8.03 (6.35–10.00)	79
Premorbid AF						
2002–2012	0.36 (0.32–0.40)	336	0.14 (0.12–0.17)	128	5.68 (4.94–6.51)	208
2002–2007	0.35 (0.29–0.40)	158	0.15 (0.11–0.19)	64	5.56 (4.49–6.80)	94
2007–2012	0.38 (0.32–0.44)	178	0.14 (0.11–0.18)	64	5.79 (4.78–6.96)	114
2007–2009.5*	0.35 (0.28–0.44)	83	0.14 (0.09–0.19)	31	5.28 (3.95–6.93)	52
2009.5*–2012	0.40 (0.33–0.49)	95	0.15 (0.10–0.21)	33	6.30 (4.83–8.08)	62

*2009.**5** represented the mid-point of the second 5 years of study and corresponded to 30 September 2009

AF, atrial fibrillation; PVE, peripheral embolic vascular event.

Of the 454 patients with AF-related incident ischaemic stroke and PVE, 436 (96%) had non-valvular AF, 129 (28.4%) had paroxysmal AF and 325 (71.6%) had permanent AF (see online supplementary tables S6–7). The characteristics of patients with AF-related ischaemic stroke and PVE were broadly similar (see online supplementary table S8a–d). Patients with AF-related incident ischaemic stroke had higher prevalence of vascular risk factors and usage of secondary preventative medications compared to those with non-AF-related stroke (table 1). In addition, AF-related strokes were also less likely to be minor (NIHSS<5): NIHSS≥10 vs 0–4; OR=3.18, 2.32 to 4.36, p<0.0001. Consequently, of 597 incident ischaemic strokes that were fatal or disabling at 6 months follow-up, 262/597 (43·9%) were AF-related (age-adjusted OR=2.52, 1.87 to 3.41, p<0.0001) and 165 (27·6%) occurred in non-anticoagulated patients with known prior AF. Of the 369 incident ischaemic strokes at age ≥80 that were fatal or disabling at 6 months, 124 (33.6%) occurred in non-anticoagulated patients with known prior AF. Of those who survived at 6 months, the AF group had higher disability (mRS 3–5) than the non-AF group (49.6% vs 28.9%; age-adjusted OR=1.84, 1.34 to 2.53, p=0.0002).

**Figure 3:**
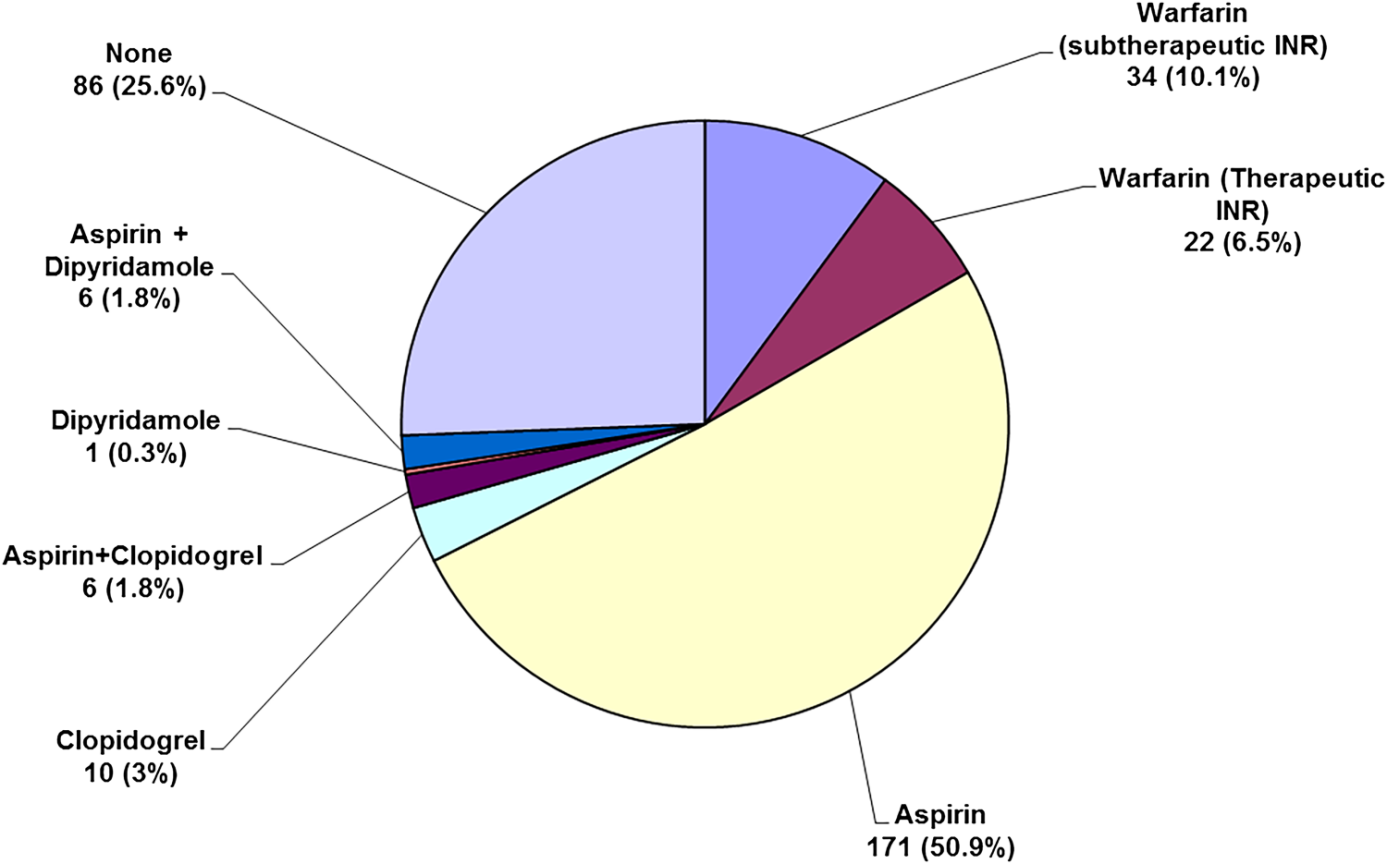
Premorbid antithrombotic therapy in the 336 OXVASC patients with incident atrial fibrillation (AF)-related ischaemic stroke or peripheral embolic vascular event and known prior AF

Among the 336 patients with incident ischaemic stroke or PVE, and known prior AF (figure 3), only 56 (16.7%) were anticoagulated (46/274 stroke and 10/62 PVE), with no increase in rates since 2007 (32/178 vs 24/158 in 2002–2007, p=0.49). Rates were higher (p=0.02) for persistent/permanent AF (46/233) than for paroxysmal AF (10/103). Of 56 patients who were anticoagulated, the International Normalised Ratio was subtherapeutic in 34 (60.7%).

Of the 280 patients with ischaemic stroke or PVE and not anticoagulated, 217 (77·5%) had a CHADS_2_ score ≥2 (see online supplementary tables S9–12), of whom 51 (23·5%) had a documented absolute or relative contraindication (see online supplementary table S13) and 181 (83.4%) had a HAS-BLED score <3 (see online supplementary table S12). A total of 194 (57.7%) were on antiplatelet drugs, but 86 (25.6%) were on no antithrombotic agent (see online supplementary table S9, figure 3). Anticoagulation was associated with reduced severity of stroke (NIHSS: anticoagulated=5.5±5.7; antiplatelet/none=8.3±7.5, p=0.006) and lower proportion of major disabled or fatal ischaemic stroke (16/69 vs 114/285, p=0.01).

Rates of premorbid anticoagulation for known prior AF in patients with AF-related ischaemic stroke or PVE were highest at younger ages, falling to 12.9% (19/147) at 80–89 and 0% (0/61) at ≥90 (see online supplementary table S9). Of the 208 patients aged ≥80 years, 189 (90.9%) were not anticoagulated but the majority with hypertension (148/168) and hypercholesterolaemia (38/57) were treated. Of these 189, 167 (88·4%) had a premorbid CHADS_2_ score ≥2, 139 (83.2%) had a HAS-BLED score <3 (see online supplementary table S12), and only 10 (5.3%) had previously had a trial of anticoagulation and had discontinued treatment (see online supplementary table S14). Of the 167 with a CHADS_2_ score ≥2, only 43 (25.7%) had any documented relative or absolute contraindication (see online supplementary table S13).

Of the 189 patients who were aged ≥80 and not anticoagulated (see online supplementary table S15a, b), 166 (87.8%) had no major disability (ie, were still independently mobile—mRS≤3) prior to the event and 99 (52.4%) were previously completely independent (mRS≤2). Of these 99 patients, 73 (73.7%) were dead or disabled 6-months postevent. Of the 167 (88·4%) who had an embolism risk score favouring treatment (CHADS_2_ score ≥2), 125 (74.9%) were dead or institutionalised after the event. Indeed, of all disabling or fatal events at age ≥80 in the study population, 230/449 (51.2%) were AF-related and 181 (40.3%) occurred in patients with known prior AF. Of 136 patients aged ≥80 with known prior AF and CHADS_2_ ≥2 who had an incident event resulting in death or institutionalisation at 6-months follow-up, only 11 (8·1%) were anticoagulated prior to the event (see online supplementary figure S1a–f). Of 128 patients with an incident intracerebral haemorrhage, 19 had known prior AF, of whom 12 were on warfarin (4 patients aged ≥80 years). The numbers of potentially preventable embolic events outnumbered warfarin-related intracerebral haemorrhages by about 15-fold (280 vs 19) at all ages and 50-fold (189 vs 4) at age ≥80 years.

## Discussion

We have made several observations that have important implications for improving prevention of stroke and other embolic events in patients with AF. First, AF was associated with 32% of all incident ischaemic strokes and PVEs. Second, one-third of all disabling or fatal ischaemic strokes at age ≥80 occurred in non-anticoagulated patients with known prior AF. Third, only 9% of patients aged ≥80 years with incident embolic events related to known prior AF were on premorbid warfarin, despite the majority having a high CHADS_2_ score and low bleeding risk, and despite the low rate of documented contraindications. Fourth, over half of those patients aged ≥80 who were not on warfarin were previously independent, but nearly three-quarters were dead or disabled 6 months after the event. Finally, we found that there was no improvement in these statistics after 2007, despite the introduction of AF registers as part of the UK Quality and Outcomes Framework (QOF) in primary care and publication of the results of the BAFTA trial on the safety and effectiveness of anticoagulation in the elderly.

The reasons for the apparent lack of impact of the QOF and the BAFTA trial results are uncertain. Of 454 patients with incident AF-related ischaemic strokes or PVEs, 118 (26%) patients did not have documented prior AF and were not aware of the diagnosis. However, a significant proportion of this apparently undocumented AF would have been either very recent or potentially have been induced by the ischaemic event itself, and this proportion did not change after 2007. In addition, the QOF rewarded primary care physicians equally for use of anticoagulants or antiplatelet agents in patients with AF prior to April 2012, despite the very substantial difference in effectiveness and only 6 of 27 available points related to AF in the latest QOF promote anticoagulation (see online supplementary table S2). The 2014 NICE guideline for AF strongly recommends using anticoagulation instead of antiplatelet agents, but did not place any particular emphasis on anticoagulation in the elderly.^23^

We found relatively high rates of premorbid antiplatelet drug use in older patients with known prior AF (table 1), reflecting evidence that physicians tended to overestimate the bleeding risks of warfarin and overestimate the benefits of antiplatelet drugs in AF.^12^ Even though the median CHADS_2_ (2–3), CHA_2_DS_2_VASc (4–5) and HAS-BLED (1–2) scores were relatively low among OXVASC patients with ischaemic event and known prior AF (probably reflecting the overall low risk for bleeding complications in this population), the embolic risk was consistently higher than the bleeding risk among those at ≥80 years (see online supplementary table S10). The substantial benefit of anticoagulants over antiplatelet drugs in high-risk patients with AF is maintained at older ages,^15^ ^24^ but the anticoagulation rate at age ≥80 in the UK ranges from only 21% to 46% (see online supplementary table S1) and studies of physician attitudes and practice show a continuing reticence to prescribe warfarin to healthy elderly patients with AF^25^ in addition to prescriptions not being tailored to AF patients’ risk factor profiles and risk scores.^26–28^ Use of anticoagulation might be increased by the availability of new oral agents, which postdated our study period, but there is currently little evidence of any increase in overall rates of anticoagulation in older age groups.^29^ ^30^

The low proportion of OXVASC patients with ischaemic stroke and known prior AF who were on premorbid oral anticoagulant is similar to that in a recent time trend study in France from 1985 to 2006.^31^ All these non-anticoagulated OXVASC patients could still represent potentially preventable strokes even though a substantial proportion of them have already received other preventative medication(s) to address various risk factors before stroke onset. In addition, the numbers of potentially preventable embolic events outnumbered warfarin-related intracerebral haemorrhages by about 15-fold at all ages and 50-fold at age ≥80 years. This finding indirectly supports observations from large cohort studies that the net clinical benefit favoured anticoagulation for almost all patients with AF at the population level except for those with very low embolic risk.^32^ ^33^

The high proportion (31%) of ischaemic stroke associated with AF in our study is consistent with other recent population-based studies despite differences in definition of AF-related stroke (see online supplementary figure S2).^34–36^ Our results also show that major fatal/disabling ischaemic strokes predominate among elderly patients with AF-related cerebral events (figure 2) and in addition, confirm findings of earlier studies regarding the severity of AF-related strokes and related consequences of greater disability, increased likelihood of subsequent institutionalisation and higher mortality rate.^34^ ^37^

Our study has several limitations. First, our findings cannot necessarily be generalised to other populations or healthcare systems. For example, rates of prior anticoagulation in patients with incident ischaemic stroke in OXVASC are somewhat lower than those in other recent stroke incidence studies (see online supplementary figure S2). However, under-use of anticoagulants is widely documented in all countries in which it has been studied, population ageing is widespread and other studies show that a high proportion of AF-related strokes occur at ages over 80 years.^31^ Second, about a quarter of our patients aged ≥80 with known prior AF had paroxysmal AF. However, the anticoagulation rate was low for both paroxysmal and permanent AF. Third, the lack of a documented reason in primary care or hospital records for patients with known prior AF for not being on warfarin might reflect poor documentation rather than under-treatment. However, the small number of patients who had been on warfarin previously but had discontinued its use was consistent with under-anticoagulation, as were the relatively low HAS-BLED scores in the majority of untreated patients, and our findings are consistent with previous studies. Fourth, although our retrospective calculation of the embolic and bleeding risk scores might have introduced some inaccuracies, information obtained from patients was cross-referenced with both hospital and primary care records. Fifth, not all vascular events in patients with AF would have been prevented by prior anticoagulation. However, warfarin reduces the rate of ischaemic stroke and PVE in primary prevention in AF by about 70% compared with placebo,^6^ ^7^ and so most of the events in untreated patients would have been preventable. In keeping with this conclusion, we found an alternative aetiology for stroke in only a small proportion of cases (see online supplementary table S7). Finally, evidence often takes several years to change clinical practice and even then, the impact of any changes on risk of vascular events would be further delayed. Moreover, we did not have individual patient data on anticoagulation rates in our underlying study population without vascular events. However, the rate of AF-related vascular events was higher in 2010–2012 than in 2007–2009.

In conclusion, a third of all fatal or disabling ischaemic strokes in people aged ≥80 years in Oxfordshire occur in non-anticoagulated patients with known prior AF and there has been no reduction in this rate since introduction of the QOF and publication of the BAFTA trial. Improved prevention in older people with AF should be a major public health priority.

## Supplementary Material

Web supplement
