# Supplementary material for: Recent time trends in incidence, outcome and premorbid treatment of atrial fibrillation-related stroke and other embolic vascular events: a population-based study
Source: J Neurol Neurosurg Psychiatry. 2015 Oct 20;88(1):12–8. doi: 10.1136/jnnp-2015-311947 (PMC5256147; doi:10.1136/jnnp-2015-311947)

Online only supplementary material

Recent time trends in incidence, outcome and pre-morbid treatment of atrial fibrillation-related stroke and other embolic vascular events: a population-based study

Gabriel S.C. Yiin, Dominic P.J. Howard, Nicola L.M. Paul, Linxin Li, Ziyah Mehta, Peter M. Rothwell on behalf of the Oxford Vascular Study

Supplementary	Title	Page
Table S1a-c	Anticoagulation rate by age and risk scores (UK and other countries)	2-7
Table S2	Quality and Outcomes Framework guidance on AF management from 2006-2014	8
S3	Additional OXVASC methodology	9-12
Table S4	Age-specific rates per 1000 population per year of first ever AF-related incident ischaemic stroke or PVE in OXVASC	13
Table S5a-b	Age-specific rates of AF-related incident ischaemic stroke or PVE at different time periods	14-15
Table S6	Distribution of AF/flutter types with timing of ischaemic stroke presentation	16
Table S7	OCSP- and TOAST- classification of AF-related ischaemic strokes	17
Table S8a-d	Baseline characteristics of patients with AF-related event	18-21
Table S9	Relationship between premorbid antithrombotic therapy, pre-morbid $CHADS_2$ score and age of onset of incident AF-related ischaemic stroke or PVE	22
Table S10	Relationship between premorbid antithrombotic therapy, pre-morbid risk scores and age in patients with incident ischaemic stroke or PVE with known prior AF	23
Table S11a-b	Premorbid warfarin use according to premorbid $CHADS_2$, CHA_2DS_2VASc and HAS-BLED scores in OXVASC patients with ischaemic stroke and PVE and known prior AF	24-25
Table S12	Comparison of premorbid $CHADS_2$ and HAS-BLED scores in patients with incident ischaemic stroke or PVE and known prior AF but not on anticoagulation	26-27
Table S13	Reasons for no premorbid warfarin use in patients at all ages and at age ≥80 years with incident ischaemic stroke or PVE, known prior AF and $CHADS_2$ score ≥2	28
Table S14	Reasons for stopping premorbid warfarin at any point prior to an embolic event	29
Table S15a-b	Changes in disability status (modified Ranking scale – mRS) in patients with an incident ischaemic stroke or PVE and any/prior AF but not on premorbid anticoagulants	30-31
Figure S1a-f	Premorbid warfarin use in OXVASC patients with known prior AF that suffered a fatal/disabling ischaemic stroke or PVE or were institutionalised at 6 months follow-up.	32-37
Figure S2	Rates of prior anticoagulation in patients with incident ischaemic stroke in OXVASC and other recent stroke incidence studies	38

Supplementary table S1a: Anticoagulation rate by age (UK studies)

Studies	Anticoagulation rate (%) by age
Bromley Hospital NHS Trust, UK (study period not specified)[1]<br>Chronic AF only	Age 75-89: 6.4%
Northumberland, UK[2]<br>Study period not specified	Age ≥75: 16.9%
General Practice Research Database (GPRD), England & Wales[3]<br>Prevalence 1998: 1.2%	1994 Age ≥75: 10.9%<br>Age ≥85: 4.5%<br>1998 Age ≥75: 21.2%<br>Age ≥85: 11.0%
ECHOES study, UK[4]<br>Prevalence 1999: 2%	Age ≥75: 17.9%
Southwest Scotland[5]<br>No prevalence given<br>27 practices, 1999	Age ≥75: 29.0%
South Wales[6,7]<br>Prevalence 2000: 1.4%<br>(6,108/424,000)	Age >75: 27.3%<br>Age >80: 20.8%
DIN-Link database, UK[8]<br>"Active" AF Prevalence 2003:1.2%<br>(12,353/1,003,372)	Age ≥75: 44.2%<br>Age ≥85: 27.1%
South London (2004), UK[9]<br>Prevalence: 1.2%<br>(944/81,811)	Age ≥75: 35.5%<br>Age ≥85: 26.4%

Supplementary table S1b: Anticoagulation rate by age (other countries)

Studies	Anticoagulation rate (%) by age
Connecticut Hospitals, USA[10] (1994)	Age 75-84: 34%<br>Age ≥85: 23%
Long term care facilities, USA[11] (1993-5)	Age 75-84: 34.6%<br>Age ≥85: 53.1%
Cardiovascular Health Study, USA (1995)[12]	Age 80-89: 24.2%<br>Age ≥90: 15.4%
Missouri Medicare beneficiaries, USA (1993-6)[13]<br>Without contraindications*	All AF patients<br>Age >75: 29%<br>Age >75: 41%*
Kaiser Permanente North California[14,15]<br>Prevalence 1996-97: 0.95% (17,955/1,890,000); without contraindications*	Age 75-84: 57.3%*<br>Age ≥85: 35.4%*
Åkersberga Community Health Centre, Sweden[16]<br>Prevalence (1992-8): 0.45%	1992-3<br>Age ≥75: 26.0%<br>1997-8<br>Age ≥75: 25.0%
Italian teaching hospital (1999)[17]<br>Prevalence:7.2%<br>Chronic AF only	Age ≥75: 11.3%
National Ambulatory National Medical Care survey, USA[18]<br>Prevalence (1991-2000): 0.57%	1991-1992<br>Age ≥80: 14.3%<br>1999-2000<br>Age ≥80: 47.5%
Stockholm South General Hospital (2002), Sweden[19]<br>*Warfarin in AF patients without contraindication	Age ≥80: 30%<br>Age ≥80: 46%*

FALSTAF Study group, France (2002)[20]<br>Permanent AF only	Age ≥80: 63.5%
National Anticoagulation Benchmark and Outcomes Report (NABOR) program, USA (2002)[21]	Age ≥80: 46%
Hospital study, USA (2001-2)[22]	Age 80-89: 45%<br>Age ≥90: 24%

Supplementary table S1c: Anticoagulation rate by age and risk scores in the UK

Studies	Anticoagulation rate (%) by $CHADS_2$ or CHA_2DS_2VASc ≥2	Anticoagulation rate (%) by age
Oxfordshire, UK (unpublished)<br>Prevalence on 31/08/2012: 1.4%<br>(9,449/676,395)	58.3%<br>(31/03/2011-31/08/2012)	--
Continuous Recording Scheme, Scotland[23]<br>Prevalence 2002: 0.87%<br>(3,135/362,155)	42%	Age ≥75: 37.1%<br>Age >85: 23.0%
GPRD, UK[24]<br>No prevalence given<br>Unclear study period<br>Chronic AF only	22.6%	Age ≥75: 32.9%<br>Age ≥80: 25.0%<br>Age >85: 15.9%
GPRD, UK[25]<br>No prevalence given<br>2000-9	$CHADS_2$ ≥2 + age ≥80: 33%<br>CHA_2DS_2VASc ≥2 + age ≥80: 32%	Age ≥80: 31.9%
GRASP-AF, UK[26]<br>Prevalence 2009-12: 1.76%<br>(231,833/13,100,000)	$CHADS_2$ ≥2 + age ≥80: 47.4%	Age ≥80:46.0%

1. Perez I, Melbourn A, Kalra L. Use of antithrombotic measures for stroke prevention in atrial fibrillation. Heart. 1999;82:570-74
2. Sudlow M, Thomson R, Thwaites B, et al. Prevalence of atrial fibrillation and eligibility for anticoagulants in the community. Lancet. 1998;352:1167-71
3. Majeed A, Moser K, Carroll K. Trends in the prevalence and management of atrial fibrillation in general practice in England and Wales, 1994-1998: analysis of data from the general practice research database. Heart. 2001;86:284-88
4. Davis RC, Hobbs FDR, Kenkre JE, et al. Prevalence of atrial fibrillation in the general population and in high-risk groups: the ECHOES study. Europace. 2012;14:1553-59

5. Burton C, Isles C, Norrie J, et al. The safety and adequacy of antithrombotic therapy for atrial fibrillation: a regional cohort study. Br J Gen Pract. 2006;56:697-702
6. Jones M, McEwan P, Morgan CL, et al. Evaluation of the pattern of treatment, level of anticoagulation control, and outcome of treatment with warfarin in patients with non-valvular atrial fibrillation: a record linkage study in a large British population. Heart. 2005;91:472-77
7. Currie CJ, Jones M, Goodfellow J, et al. Evaluation of survival and ischaemic and thromboembolic event rates in patients with non-valvular atrial fibrillation when treated and untreated with warfarin. Heart. 2006;92:196-200
8. De Wilde S, Carey IM, Richards N, et al. Trends in the prevalence of diagnosed atrial fibrillation, its treatment with anticoagulation and predictors of such treatment in UK primary care. Heart. 2006;92:1064-70
9. De Lusignan S, Van Vlymen J, Hague N, et al. Preventing stroke in people with atrial fibrillation: a cross sectional study. J Public Health. (Oxf) 2005;27:85-92
10. Brass LM, Krumholz HM, Scinto JM, et al. Warfarin use among patients with atrial fibrillation. Stroke. 1997;28:2382-9
11. Gurwitz JH, Monette J, Rochon PA, et al. Atrial fibrillation and stroke prevention with warfarin in the long term care setting. Arch Intern Med. 1997;157:978-84
12. White R, McBurnie MA, Manolio T, et al. Oral anticoagulation in patients with atrial fibrillation: adherence with guidelines in an elderly cohort. Am J Med. 1999;106:165-71
13. Gage BF, Boechler M, Doggette AL, et al. Adverse outcomes and predictors of underuse of antithrombotic therapy in Medicare Beneficiaries with chronic atrial fibrillation. Stroke. 2000;31:822-27
14. Go AS, Hylek EM, Phillips KA, et al. Prevalence of diagnosed atrial fibrillation in adults: National implications for rhythm management and stroke prevention: The AnTicoagulation and Risk Factors in Atrial Fibrillation (ATRIA) Study. JAMA. 2001;285:2370-75
15. Go AS, Hylek EM, Borowsky LH, et al. Warfarin use among ambulatory patients with non-valvular atrial fibrillation: the AnTicoagulation and risk factors in Atrial Fibrillation (ATRIA) Study. Ann Intern Med. 1999;131:927-34
16. Wandell PE. Drug use in patients with atrial fibrillation in Swedish primary health care: a comparison 5 years apart. Eur J Clin Pharmacol. 1999;55:333-7
17. Ageno W, Ambrosini F, Nardo B, et al. Atrial fibrillation and antithrombotic treatment in Italian hospitalized patients: a prospective, observational study. J Thrombo Thrombolysis. 2001;12:225-30
18. Fang MC, Stafford RS, Ruskin JN, Singer DE. National trends in antiarrhythmic and antithrombotic medication use in atrial fibrillation. Arch Intern Med. 2004;164:55-60
19. Friberg L, Hammar N, Ringh M, et al. Stroke prophylaxis in atrial fibrillation: who gets it and who does not? Report from the Stockholm Cohort-study on Atrial Fibrillation (SCAF-study). Eur Heart J. 2006;27:1954-64
20. FALSTAF Study Group, Leizorovicz A, Cohen A, Guenoun M, et al. Influence of age on the prescription of vitamin K antagonists in outpatients with permanent atrial fibrillation in France. Pharmacoepidemiol Drug Saf. 2007;16:32-8

21. Waldo AL, Becker RC, Tapson VF, et al. Hospitalized patients with atrial fibrillation and a high risk of stroke are not being provided with adequate anticoagulation. J Am Coll Cardiol. 2005;46:1729-36
22. Hylek EM, D'Antonio J, Evans-Molina C, et al. Translating the results of randomized trials into clinical practice: the challenge of warfarin candidacy among hospitalized elderly patients with atrial fibrillation. Stroke. 2006;37:1075-80
23. Murphy NF, Simpson CR, Jhund PS, et al. A national survey of the prevalence, incidence, primary care burden and treatment of atrial fibrillation in Scotland. Heart. 2007;93:606-12
24. Rietbrock S, Plumb JM, Gallagher AM, et al. How effective are dose adjusted warfarin and aspirin for the prevention of stroke in patients with chronic atrial fibrillation? An analysis of the UK General Practice Research Database. Thromb Haemost. 2009;101:527-34
25. Scowcroft ACE, Lee S, Mant J. Thrombophylaxis of elderly patients with AF in the UK: an analysis using the General Practice Research Database (GPRD) 2000-2009. Heart. 2013; 99(2):127-32.
26. Cowan C, Healicon R, Robson I, et al. The use of anticoagulants in the management of atrial fibrillation among general practices in England. Heart. 2013;99:1166-72.

Supplementary table S2: Quality and Outcomes Framework guidance on AF management from 2006-2014[§]

Indicator	Points	2006-7	2007-8	2008-9	2009-10*	2010-11*	2011-12*	2012-13*	Payment stages
AF register (2006-2014)	5	Yes	Yes	Yes	Yes	Yes	Yes	Yes	
AF diagnosis (2006-12)	10	Yes	Yes	Yes	Yes	Yes	Yes	--	40-90%
OAC or antiplatelet agents (2006-12)	15	Yes	Yes	Yes	**points ↓from 15 to 12**	**points ↓from 15 to 12**	**points ↓from 15 to 12**	see below	40-90%
Use of $CHADS_2$ in preceding 15 months (2012-13)[α]	10	--	--	--	--	--	--	Yes	40-90%
$CHADS_2$ score =1 in preceding 15 months + use of OAC or antiplatelet (2012-13)[α]	6	--	--	--	--	--	--	Yes	50-90%
$CHADS_2$ ≥2 and treated with OAC (2012-13)[β]	6	--	--	--	--	--	--	Yes	40-70%

[§]http://www.qof.ic.nhs.uk. Accessed December 10th 2014.
*Maximum QOF points for AF reduced from 30 in 2006-2009 to 27 in 2009-2013.
[α]12 instead of 15 months for 2013-2014.
[β]$CHADS_2$ ≥1 instead of ≥2 for 2013-2014.

Supplementary S3

OCSP and OXVASC Methodology

OXVASC is based on the Oxfordshire Community Stroke Project (OCSP),[1] a similarly designed population-based incidence study of stroke and TIA performed in the 1980s in the same population. The OXVASC and OCSP primary care practices had the same hospital referral patterns, held accurate age-sex registers of their patients, and were willing to refer any patient with a suspected cerebrovascular event to the study and allow regular searches of their computerised diagnostic coding systems. In both studies, the population was 94% white.[2]

Definitions and diagnosis: Diagnosis was designed to be as similar as possible in OXVASC to OCSP. The same definitions of stroke and TIA were used.[3] However, since clinical opinion about which clinical syndromes represent TIA or stroke has evolved over the last 25 years, the Principal Investigator of the OCSP had input into the design of OXVASC and reviewed a subset of cases to ensure that the application of definitions of events was comparable. Diagnosis was based on clinical findings and CT brain imaging as in OCSP. In addition, all CT scans were reviewed by a study neuroradiologist who was also involved in the OCSP and used the same criteria for haemorrhagic infarction and primary intracerebral haemorrhage as in the OCSP.

All events were categorised as first-ever incident or recurrent based on clinical history rather than findings on brain imaging. As in the OCSP, a first-ever stroke that occurred in a patient with a previous TIA was coded as incident, but a first-ever TIA in a patient with a previous stroke was coded as recurrent.[1] The other OCSP requirements for definition of an "incident" TIA were also adhered to.[1] As in the OCSP, patients who had an event whilst temporarily away from Oxford were included, but visitors to Oxford who were not registered with one of the study FPs were excluded.

Case Ascertainment: The following sources of ascertainment were used in OXVASC and OCSP:
1) Collaborating primary care physicians reported cases to the study physicians by telephone, fax or pager as soon as they became aware of a possible TIA or stroke. Patients not requiring immediate hospital admission were seen in a daily clinic, or at home if transfer to hospital was not inappropriate.
2) The study team maintained frequent personal contact with the general practices by regular visits, a quarterly newsletter, and via a liaison family physician in each practice.
3) Computerised hospital diagnostic codes were reviewed regularly. In OCSP, the Oxford Record Linkage system[4] was used. In OXVASC, the coding department for the Oxford Hospitals Trust provided a monthly GP-practice specific list of all patients with ICD-10 codes for TIA and stroke and all deaths in hospital. A similar list was obtained from the Oxford Eye Hospital and local community hospitals.
4) Hospital admission and Emergency Department registers were reviewed daily.
5) Out of hospital deaths were identified via the Coroner's Office, by review of all death certificates in the study practices, and by ICD-10 vascular death codes from the local Department of Public Health.

Several methods of case-ascertainment that were not used in OCSP were employed in OXVASC:
1) Daily visits to the Acute Medical Admissions Unit, Acute Stroke Unit, Neurology wards and Stroke Rehabilitation wards, and daily contact with Hospital Bereavement Officers to identify all patients brought into hospital dead or who died soon after arrival.
2) A computer-generated list of all requests for brain and cerebral vascular imaging was reviewed on a monthly basis and all referrals for carotid Doppler ultrasound were reviewed on a weekly basis.

3) Patients with visual symptoms were referred directly to the study from the Eye Emergency Unit and Department of Ophthalmology and lead clinical staffs in the other departments (e.g. paediatrics, obstetrics etc) were contacted monthly to ascertain strokes in patients under their care.

Clinical assessment and investigation: A study clinician assessed patients as soon as possible after the event in hospital, in a daily dedicated clinic or at home. Informed consent was sought, where possible, or assent was obtained from a relative. A standard clinical history and examination were performed. As in the OCSP, pre-morbid handicap and disability was assessed with the Rankin[5] score. If a patient died prior to assessment, an eyewitness account of the clinical event and reviewed any relevant records were obtained. Event and baseline characteristics were recorded and all patients underwent standardised investigations including blood screen, 12-lead electrocardiogram, brain imaging (CT and/or MRI) and vascular imaging (carotid/peripheral duplex ultrasonography or CT/MR angiography). The treating physicians completed supplementary investigations such as echocardiography when indicated.

If death occurred outside hospital or prior to brain imaging, the autopsy result was reviewed. Given the high rate (98%) of imaging/autopsy in OXVASC, strokes of unknown type were coded as ischaemic for the purpose of analysis. In OCSP, strokes that did not have brain imaging or autopsy (12%) were classified as haemorrhages only if clinical scoring systems indicated a high degree of certainty.[6] Otherwise they are coded as ischaemic for the purpose of this analysis. Diagnosis of subarachnoid haemorrhage[6] and clinical subtyping of stroke[7] were the same as in OCSP.

Both OCSP and OXVASC recorded pre-morbid medication and vascular risk factors from the patient or relative, hospital records and primary care records. The most recent measurement of blood pressure was recorded in both studies from the general practice records. Total cholesterol concentration was measured at the time of assessment after the TIA or stroke. All surviving cases were followed-up by a research nurse or therapist at 1, 6, 12, 24, 60, and 120 months from the time of the stroke and the modified Rankin score was determined. If a recurrent vascular event was suspected, the patient was assessed by a study physician.

1. Dennis MS, Bamford JM, Sandercock PA, et al. Incidence of transient ischemic attacks in Oxfordshire, England. Stroke. 1989;20:333-9.
2. 2001 census area statistics. London: Stationery Office, 2001
3. Hatano S. Experience from a multicentre stroke register: a preliminary report. Bull World Health Organ. 1976;54:541-53.
4. Acheson ED. Medical record linkage. London: OUP, 1967.
5. van Swieten JC, Koudstaal PJ, Visser MC, et al. Interobserver agreement for the assessment of handicap in stroke patients. Stroke. 1988;19:604-7.
6. Bamford J, Sandercock P, Dennis M, et al. A prospective study of acute cerebrovascular disease in the community: the Oxfordshire Community Stroke Project--1981-86. 2. Incidence, case fatality rates and overall outcome at one year of cerebral infarction, primary intracerebral and subarachnoid haemorrhage. Journal Neurol Neurosurg Psychiatry. 1990;53:16-22.
7. Bamford J, Sandercock P, Dennis M, et al. Classification and natural history of clinically identifiable subtypes of cerebral infarction. Lancet. 1991;337:1521-6.

Extended methods for ascertainment of peripheral vascular events (PVEs) in OXVASC

Ascertainment of patients with acute PVEs was done using overlapping methods of hot and cold pursuit similar to those used for acute cerebral events. Hot pursuit was based on: 1) Daily searches of Emergency Department admission and symptom/diagnosis registers; 2) Daily listing from the central admissions department of all patients from our general practices admitted to hospital, and assessment of these patients in hospital; 3) Daily visits to the cardiac surgery and vascular surgery wards and review of daily lists of all patients referred to vascular surgery or for peripheral angiography; 4) Daily identification via Bereavement Officers of patients dead on arrival at hospital or who died soon after; 5) Daily assessment of all patients undergoing diagnostic angiographic, angioplasty/stenting or peripheral arterial surgical procedures in any territory.

The methods of cold pursuit were: 1) Weekly review of all listed surgical procedures undertaken by vascular and cardiovascular surgery; 2) Frequent contact with general practices and monthly searches of computerised practice diagnostic codes; 3) Monthly practice-specific list of all patients with relevant diagnostic codes from the coding departments covering all acute and community hospitals; 4) Monthly visits to the Coroner's Office to review out-of-hospital deaths; 5) Review of all death certificates and relevant clinical details in the study general practices; 6) Practice-specific listings of all ICD-10 death codes from the local Department of Public Health; 7) Review of vascular surgery outpatient clinic letters to identify all patients attending vascular clinics who were not admitted to hospital.

A study clinician assessed patients as soon as possible after the event. Informed consent was sought, where possible, or assent was obtained from a relative. Standardised clinical history and cardiovascular examination were recorded. We also recorded from the patient, their hospital records and their primary care records, details of the clinical event, medication, past medical history, all investigations relevant to their admission and all interventions subsequent to the event. Vascular assessment included clinical examination and measurement of the abdominal and peripheral pulses, Buerger's test and ankle Doppler pressure recordings. For cases in which clinical vascular assessment was not possible by the study clinician prior to urgent revascularisation or death, we used assessments made by the admitting clinician.

If a patient died prior to assessment, we obtained eyewitness accounts and reviewed any relevant records. If death occurred outside hospital or prior to investigation, the autopsy result was reviewed. Clinical details were sought from primary care physicians or other clinicians on all deaths of possible vascular aetiology. Initial clinical assessments were made by study clinical research fellows alongside the clinical teams. All diagnoses were subsequently reviewed by a vascular surgeon.

Acute peripheral arterial events were defined as any acute vascular event in any part of the arterial system that affected the aorta and its major branches, a limb or an organ other than the heart or the brain/eye and led to hospital admission or caused death in the community. All patients were followed-up by a research nurse or via their family doctor, with recurrent events also identified by the on-going study surveillance. If a recurrent vascular event was suspected, the patient was assessed by a study physician. All events were categorised as first-ever incident or recurrent and specific to territory. An incident acute peripheral vascular event implied the first ever acute embolic arterial event in any vascular territory apart from the brain. Only incident events were included in the analysis for this manuscript. Patients who had an event whilst temporarily away from Oxfordshire were included, but visitors to Oxfordshire who were not registered with one of the study practices were excluded.

Recording and coding of deaths in the OXVASC population

Out of hospital death accounts for a small proportion of ischaemic strokes and PVE, but In view of previous data on the inaccuracy of death certification of vascular disease[1-2], all deaths in the OXVASC population were recorded and coded as follows:

i. *All certified deaths due to vascular disease*: All deaths with the underlying cause coded for the purposes of national statistics as being due to "ischaemic heart disease" (ICD I210-I229, I251, I259), "cerebrovascular disease" (ICD I600-I619, I630-I669, I670, I678, I679, I694, I698), "peripheral vascular disease" (ICD I700, I710-I729, I739-I749, I790), or "visceral vascular disease" (ICD D735, K763, N280, K550, K551, K558, K559).

ii. *All certified acute vascular deaths*: All deaths where the underlying cause of death was coded for the purposes of national statistics as being due to "acute ischaemic heart disease" (ICD I210-I229), "acute cerebrovascular disease" (ICD I600-I619, I630-I669), "acute peripheral vascular disease" (ICD I710, I711, I713, I715, I718, I720-I729, I739-I749, I790), or
"acute visceral vascular disease" (ICD D735, K763, N280, K550, K558, K559)

iii. *Probable or definite acute vascular deaths*: All deaths in (ii) excluding unclassifiable sudden deaths (as defined below) and other deaths that after evaluation of the clinical and/or post-mortem data were considered to have been incorrectly attributed (i.e. there was definite evidence of another cause), and with the addition of any deaths not in (ii) that were considered by the OXVASC researchers to have been due to an acute vascular event.

iv. Possible acute vascular deaths: All deaths in (iii) excluding sudden deaths for which the only supporting evidence was a previous history of symptomatic vascular disease in the relevant arterial territory (unless there was a documented acute myocardial infarction, acute stroke or acute peripheral arterial event within the previous 30 days).

Unclassifiable deaths, which were mainly out-of-hospital deaths, were certified as having a vascular cause, but had no witness description of the event or of preceding symptoms to suggest acute vascular disease in a specific territory, no post-mortem evidence of an acute vascular event or other cause of death, no post-mortem evidence of clinically significant coronary atherosclerosis, and no past history of symptomatic vascular disease in a relevant arterial territory.

1. Coull AJ, Silver LE, Bull LM, et al. Direct assessment of completeness of ascertainment in a stroke incidence study. Stroke. 2004;35:2041-5.
2. Luepker RV, Apple FS, Christenson RH, et al. Case definitions for acute coronary heart disease in epidemiology and clinical research studies: a statement from the AHA Council on Epidemiology and Prevention; AHA Statistics Committee; World Heart Federation Council on Epidemiology and Prevention; the European Society of Cardiology Working Group on Epidemiology and Prevention; Centers for Disease Control and Prevention; and the National Heart, Lung, and Blood Institute. Circulation. 2003;108:2543-9

Supplementary table S4: Age-specific rates per 1000 population per year of first ever AF-related incident ischaemic stroke or peripheral embolic vascular event (PVE) in OXVASC

Ischaemic stroke	Men	Rate per 1000 per year (95% CI)	Women	Rate per 1000 per year (95% CI)	Total	Rate per 1000 per year (95% CI)
<60	11/38736	0.03 (0.01,0.05)	1/35656	0.00 (0.00,0.02)	12/74392	0.02 (0.01,0.03)
60-69	20/4308	0.46 (0.28,0.72)	20/4332	0.46 (0.28,0.71)	40/8640	0.46 (0.33,0.63)
70-79	57/2848	2.00 (1.52,2.59)	50/3187	1.57 (1.16,2.07)	107/6035	1.77 (1.45,2.14)
80-89	72/1207	5.97 (4.67,7.51)	91/1914	4.75 (3.83,5.84)	163/3121	5.22 (4.45,6.09)
≥90	11/147	7.50 (3.74,13.41)	50/393	12.73 (9.45,16.79)	61/540	11.31 (8.65,14.53)
Total	171/47246	0.36 (0.31,0.42)	212/45482	0.47 (0.41,0.53)	383/92728	0.41 (0.37,0.46)
				**Premorbid AF**	274/92728	0.30 (0.26,0.33)
				**New AF**	109/92728	0.12 (0.10,0.14)
**PVE**						
<60	2/38736	0.01 (0.00,0.02)	0/35656	--	2/74392	0.00 (0.00,0.01)
60-69	1/4308	0.02 (0.00,0.13)	1/4332	0·02 (0.00,0.13)	2/8640	0.02 (0.00,0.08)
70-79	8/2848	0.28 (0.12,0.55)	11/3187	0·35 (0.17,0.62)	19/6035	0.31 (0.19,0.49)
80-89	11/1207	0.91 (0.46,1.63)	20/1914	1·04 (0.64,1.61)	31/3121	0.99 (0.67,1.41)
≥90	5/147	3.41 (1.11,7.95)	12/393	3·06 (1.58,5.34)	17/540	3.15 (1.84,5.05)
Total	27/47246	0.06 (0.04,0.08)	44/45482	0·10 (0.07,0.13)	71/92728	0.08 (0.06,0.10)
				**Premorbid AF**	62/92728	0.07 (0.05,0.09)
				**New AF**	9/92728	0.01 (0.00,0.02)

Supplementary table S5a: Age-specific rates of AF-related incident ischaemic stroke at different time periods

Ischaemic stroke	All Ages Rate/1000/year	Number	Age <80 Rate/1000/year	Number	Age ≥80 Rate/1000/year	Number
**Total AF**						
2002-2012	0.41 (0.37-0.46)	383	0.18 (0.15-0.21)	159	6.12 (5.34-6.98)	224
2002-2007	0.42 (0.36-0.48)	191	0.18 (0.14-0.22)	78	6.68 (5.50-8.03)	113
2007-2012	0.41 (0.35-0.47)	192	0.18 (0.14-0.22)	81	5.64 (4.64-6.79)	111
2007-2009.5*	0.36 (0.29-0.45)	85	0.16 (0.11-0.22)	36	4.98 (3.86-6.58)	49
2009.5*-2012	0.45 (0.37-0.55)	107	0.20 (0.15-0.27)	45	6.30 (4.83-8.08)	62
**Premorbid AF**						
2002-2012	0.30 (0.26-0.33)	274	0.12 (0.10-0.15)	110	4.48 (3.82-5.22)	164
2002-2007	0.28 (0.23-0.33)	127	0.13 (0.09-0.16)	55	4.26 (3.33-5.36)	72
2007-2012	0.31 (0.26-0.37)	147	0.12 (0.09-0.16)	55	4.67 (3.77-5.73)	92
2007-2009.5*	0.30 (0.24-0.38)	70	0.12 (0.08-0.17)	27	4.37 (3.16-5.88)	43
2009.5*-2012	0.33 (0.26-0.41)	77	0.12 (0.08-0.18)	28	4.98 (3.68-6.58)	49
**New AF**						
2002-2012	0.12 (0.10-0.14)	109	0.06 (0.04-0.07)	49	1.64 (1.25-2.11)	60
2002-2007	0.14 (0.11-0.18)	64	0.05 (0.03-0.08)	23	2.42 (1.74-3.29)	41
2007-2012	0.10 (0.07-0.13)	45	0.06 (0.04-0.08)	26	0.97 (0.58-1.51)	19
2007-2009.5*	0.06 (0.04-0.10)	15	0.04 (0.02-0.08)	9	0.61 (0.22-1.33)	6
2009.5*-2012	0.13 (0.09-0.18)	30	0.08 (0.04-0.12)	17	1.32 (0.70-2.26)	13

*2009.5 represented the mid-point of the second 5 years of study and corresponded to 30th September 2009

Supplementary table S5b: Age-specific rates of AF-related incident peripheral embolic vascular event (PVE) at different time periods

PVE	All Ages Rate/1000/year	Number	Age <80 Rate/1000/year	Number	Age ≥80 Rate/1000/year	Number
**Total AF**						
2002-2012	0.08 (0.06-0.10)	71	0.03 (0.02-0.04)	23	1.31 (0.97-1.74)	48
2002-2007	0.07 (0.05-0.10)	32	0.02 (0.01-0.04)	10	1.30 (0.81-1.97)	22
2007-2012	0.08 (0.06-0.11)	39	0.03 (0.02-0.05)	13	1.32 (0.86-1.94)	26
2007-2009.5*	0.06 (0.03-0.10)	14	0.02 (0.01-0.05)	5	0.91 (0.42-1.74)	9
2009.5*-2012	0.11 (0.07-0.16)	25	0.04 (0.02-0.07)	8	1.73 (1.01-2.77)	17
**Premorbid AF**						
2002-2012	0.07 (0.05-0.09)	62	0.02 (0.01-0.03)	18	1.20 (0.87-1.61)	44
2002-2007	0.07 (0.05-0.10)	31	0.02 (0.01-0.04)	9	1.30 (0.81-1.97)	22
2007-2012	0.07 (0.04-0.09)	31	0.02 (0.01-0.04)	9	1.12 (0.70-1.69)	22
2007-2009.5*	0.06 (0.03-0.09)	13	0.02 (0.00-0.05)	4	0.91 (0.42-1.74)	9
2009.5*-2012	0.08 (0.05-0.12)	18	0.02 (0.01-0.05)	5	1.32 (0.70-2.26)	13
**New AF**						
2002-2012	0.01 (0.00-0.02)	9	0.01 (0.00-0.01)	5	0.11 (0.03-0.28)	4
2002-2007	0.00 (0.00-0.01)	1	0.00 (0.00-0.01)	1	--	0
2007-2012	0.02 (0.01-0.03)	8	0.01 (0.00-0.02)	4	0.20 (0.06-0.52)	4
2007-2009.5*	0.00 (0.00-0.02)	1	0.01 (0.00-0.02)	1	--	0
2009.5*-2012	0.03 (0.01-0.06)	7	0.01 (0.00-0.04)	3	0.41 (0.11-1.04)	4

*2009.5 represented the mid-point of the second 5 years of study and corresponded to 30th September 2009

Supplementary table S6: Distribution of AF/flutter types with timing of ischaemic stroke presentation in OXVASC

	Paroxysmal	Persistent*	Permanent	Total
Premorbid				
<80 years	36	20	54	110
≥80 years	46	28	90	164
Only at presentation	12	53	10	75
Within first month	17	13	4	34
**Total**	111	114	158	383

*AF was defined as persistent rather than permanent if it had been present apparently continuously but for less than one year

Supplementary table S7: The Oxford and TOAST-classification of AF-related ischaemic strokes in OXVASC

Oxford-classification

**Category**	**Number (%)**
Total anterior circulation infarct	86 (22.5)
Partial anterior circulation infarct	158 (41.3)
Lacunar infarct	39 (10.2)
Posterior circulation infarct	92 (24.0)
Anterior and posterior circulation	4 (1.0)
Uncertain	4 (1.0)
**Total**	383 (100)

TOAST-classification

**Category**	**Paroxysmal AF Known (%)**	**Paroxysmal AF New (%)**	**Persistent/ Permanent AF (%)**	**Total (%)**
Cardioembolic	74 (90.2)	23 (79.3)	248 (91.1)	345 (90.1)
Large vessel	0 (0)	0 (0)	3 (1.1)	3 (0.8)
Small vessel	0 (0)	0 (0)	0 (0)	0 (0)
Undetermined	0 (0)	0 (0)	0 (0)	0 (0)
Unknown	0 (0)	0 (0)	0 (0)	0 (0)
Multiple[@]	8 (9.8)	6 (20.7)	21 (7.7)	35 (9.1)
Other	0 (0)	0 (0)	0 (0)	0 (0)
**Total**	82 (100)	29 (100)	272 (100)	383 (100)

[@]"Multiple causes" group related to the presence of AF and symptomatic (≥50%) carotid/vertebrobasilar stenosis or lacunar syndrome in patients with ischaemic stroke

Supplementary table S8a-d: Baseline characteristics of patients with AF-related event

Supplementary table S8a: Baseline characteristics of patients with AF-related incident peripheral embolic vascular event (PVE) or ischaemic stroke

	PVE (n=71)	Ischaemic stroke (n=383)	P value
Male sex (%)	27 (38.0)	171 (44.6)	0.30
Mean age (SD)	82.5 (9.8)	80.0 (9.7)	0.05
Congestive cardiac failure	25 (35.2)	99 (25.8)	0.10
Hypertension	55 (77.5)	287 (74.9)	0.507
Diabetes	10 (14.1)	58 (15.1)	0.82
Previous TIA	11 (11.5)	60 (15.7)	0.97
Previous MI	20 (28.2)	72 (18.8)	0.07
Angina	16 (22.5)	102 (26.6)	0.47
Current smoking	11 (15.5)	30 (7.8)	0.039
Hypercholesterolaemia[#]	30 (42.3)	118 (30.8)	0.06
Valvular heart disease	22 (31.0)	81 (21.4)	0.08
Venous thromboembolism	6 (8.5)	27 (7.1)	0.68
Antiplatelet agent(s)	37 (52.1)	202 (52.7)	0.92
Lipid lowering agent	27 (38.0)	102 (26.6)	0.05
Antihypertensive(s)	56 (78.9)	282 (73.6)	0.35
Anticoagulant	10 (14.1)	47 (12.3)	0.67

[#]defined as ≥6.0 mmol/l

Supplementary table S8b: Baseline characteristics of patients at age ≥80 with AF-related incident peripheral embolic vascular event (PVE) or ischaemic stroke

	PVE (n=48)	Ischaemic stroke (n=224)	P value
Male sex (%)	16 (33.3)	83 (37.1)	0.63
Mean age (SD)	87.7 (4.2)	86.6 (4.3)	0.10
Congestive cardiac failure	20 (41.7)	60 (26.8)	0.040
Hypertension	36 (75.0)	178 (79.5)	0.49
Diabetes	7 (14.6)	28 (12·5)	0.70
Previous TIA	8 (16.7)	39 (17.4)	0.90
Previous MI	14 (29.2)	46 (20.5)	0.19
Angina	10 (20.8)	68 (30.4)	0.19
Current smoking	4 (8.3)	5 (2.2)	0.06
Hypercholesterolaemia[#]	13 (27.1)	59 (26.3)	0.92
Valvular heart disease	17 (35.4)	48 (21.4)	0.039
Venous thromboembolism	6 (12.5)	14 (6.2)	0.14
Antiplatelet agent(s)	25 (52.1)	127 (56.7)	0.56
Lipid lowering agent	11 (22.9)	54 (24.1)	0.86
Antihypertensive(s)	37 (77.1)	174 (77.7)	0.93
Anticoagulant	5 (10.4)	15 (6.7)	0.37

[#]defined as ≥6.0 mmol/l

Supplementary table S8c: Baseline characteristics of patients at all ages with incident peripheral embolic vascular event (PVE) or ischaemic stroke related to known premorbid AF (N=336)

	PVE (n=62)	Ischaemic stroke (n=274)	P value
Male sex (%)	24 (38.7)	126 (46.0)	0.30
Mean age (SD)	83.4 (8.2)	80·4 (9.4)	0.019
Congestive cardiac failure	23 (37.1)	85 (31.0)	0.36
Hypertension	47 (75.8)	212 (77.4)	0.79
Diabetes	9 (14.5)	49 (17.9)	0.53
Previous TIA	10 (16.1)	46 (16.8)	0.90
Previous MI	19 (30.6)	59 (21.5)	0.13
Angina	15 (24.2)	87 (31.8)	0.24
Current smoking	7 (11.3)	17 (6.2)	0.17
Hypercholesterolaemia[#]	24 (38.7)	88 (32.1)	0.32
Valvular heart disease	20 (32.3)	73 (26.6)	0.37
Venous thromboembolism	6 (9.7)	21 (7.7)	0.61
Antiplatelet agent(s)	33 (53.2)	167 (60.9)	0.26
Lipid lowering agent	21 (33.9)	82 (29.9)	0.54
Antihypertensive(s)	48 (77.4)	214 (78.1)	0.91
Anticoagulant	10 (16.1)	46 (16·8)	0.90

[#]defined as ≥6.0 mmol/l

Supplementary table S8d: Baseline characteristics of patients at age ≥80 with incident peripheral embolic vascular event (PVE) or ischaemic stroke related to known prior AF (N=208)

	PVE (n=44)	Ischaemic stroke (n=164)	P value
Male sex (%)	15 (34.1)	61 (37.2)	0.70
Mean age (SD)	87.7 (4.2)	86.6 (4.3)	0.13
Congestive cardiac failure	19 (43.2)	52 (31·7)	0.15
Hypertension	32 (72.7)	136 (82.9)	0.13
Diabetes	7 (15.9)	27 (16.5)	0.93
Previous TIA	8 (18.2)	30 (18.3)	0.99
Previous MI	14 (31.8)	37 (22.6)	0.21
Angina	9 (20.5)	58 (35.4)	0.06
Current smoking	4 (9.1)	1 (0.6)	0.008
Hypercholesterolaemia[#]	11 (25.0)	46 (28.0)	0.69
Valvular heart disease	16 (36.4)	41 (25.0)	0.13
Venous thromboembolism	6 (13.6)	9 (5.5)	0.09
Antiplatelet agent(s)	24 (54.5)	108 (65.9)	0.17
Lipid lowering agent	9 (20.5)	46 (28.0)	0.31
Antihypertensive(s)	32 (72.7)	135 (82.3)	0.27
Anticoagulant	5 (11.4)	14 (8.5)	0.56

[#]defined as ≥6.0 mmol/l

Supplementary table S9: Relationship between premorbid antithrombotic therapy, pre-morbid $CHADS_2$ score and age of onset of incident AF-related ischaemic stroke or peripheral embolic vascular event

Age group	Warfarin<br>n, (%)	No anti-thrombotics<br>n, (%)	Mono-antiplatelet<br>n, (%)	Dual-antiplatelet<br>n, (%)
**<60**	5 (55.6)	1 (11.1)	3 (33.3)	0
**60-69**	8 (28.6)	7 (25.0)	13 (46.4)	0
**70-79**	24 (26.4)	19 (20.9)	44 (48.4)	4 (4.4)
**80-89**	19 (12.9)	42 (28.6)	83 (56.5)	3 (2.0)
**≥90**	0	17 (27.9)	39 (63.9)	5 (8.2)
**Total**	56 (16.7)	86 (25.6)	182 (54.2)	12 (3.6)
**$CHADS_2$ <2**				
**<60**	1 (20.0)	1 (20.0)	3 (60.0)	0
**60-69**	6 (33.3)	4 (22.2)	8 (44.4)	0
**70-79**	6 (19.4)	6 (19.4)	19 (61.3)	0
**80-89**	3 (17.6)	8 (47.1)	5 (29.4)	1 (5.9)
**≥90**	0	3 (37.5)	4 (50.0)	1 (12.5)
**Total**	16 (20.3)	22 (27.8)	39 (49.4)	2 (2.5)
**$CHADS_2$ ≥2**				
**<60**	4 (100)	0	0	0
**60-69**	2 (20.0)	3 (30.0)	5 (50.0)	0
**70-79**	18 (30.0)	13 (21.7)	25 (41.7)	4 (6.7)
**80-89**	16 (12.3)	34 (26.2)	78 (60.0)	2 (1.5)
**≥90**	0	14 (26.4)	35 (66.0)	4 (7.5)
**Total**	40 (15.6)	64 (24.9)	143 (55.6)	10 (3.9)

Supplementary table S10: Relationship between premorbid antithrombotic therapy, pre-morbid risk scores and age in patients with incident ischaemic stroke or peripheral embolic vascular event (PVE) with known prior AF in OXVASC

	$CHADS_2$ Mean (Median)	CHA_2DS_2VASc Mean (Median)	HAS-BLED* Mean (Median)	Warfarin [n (%)]	No anti-thrombotics [n (%)]	Mono-antiplatelet [n (%)]	Dual-antiplatelet [n (%)]
**Ischaemic stroke**							
**Age group**							
<60 (n=8)	1.38 (1.5)	1.88 (1.5)	0.75 (1)	5 (62.5)	1 (12.5)	2 (25)	0
60-69 (n=27)	1.41 (1)	2.67 (2)	1.33 (1)	7 (25.9)	7 (25.9)	13 (48.1)	0
70-79 (n=75)	2.01 (2)	3.76 (4)	1.77 (2)	20 (26.7)	15 (20)	38 (50.7)	2 (2.7)
80-89 (n=118)	2.64 (2·5)	4.57 (4)	1.47 (1)	14 (11.9)	32 (27.1)	69 (58.5)	3 (2.5)
≥90 (n=46)	2.74 (2·5)	4.80 (5)	1.65 (2)	0	11 (23.9)	33 (71.7)	2 (4.3)
Total	2.32 (2)	4.12 (4)	1.55 (1)	46 (16.8)	66 (24.1)	155 (56.6)	7 (2.6)
**PVE**							
**Age group**							
<60 (n=1)	0	0	1 (1)	0	0	1 (100)	0
60-69 (n=1)	1.00 (1.00)	3.00 (3.0)	1 (1)	1 (100)	0	0	0
70-79 (n=16)	2.31 (2.5)	4·56 (4.5)	2.19 (2)	4 (25.0)	4 (25.0)	6 (37.5)	2 (12.5)
80-89 (n=29)	3.21 (3)	5·48 (6)	2.34 (2)	5 (17.2)	10 (34.5)	14 (48.3)	0
≥90 (n=15)	2.40 (2)	4·67 (5)	2.20 (2)	0	6 (40.0)	6 (40·0)	3 (20.0)
Total	2.69 (3)	4.92 (5)	2.23 (2)	10 (16.1)	20 (32.3)	27 (43.5)	5 (8.1)

*We did not have information on labile INR in those patients on pre-morbid warfarin and hence the maximum HAS-BLED score was out of eight instead of nine.

Supplementary table S11a-b: Premorbid warfarin use according to premorbid $CHADS_2$, CHA_2DS_2VASc and HAS-BLED scores in patients with incident ischaemic stroke (11a) and peripheral embolic vascular event (PVE) (11b) and known prior AF in OXVASC.

S11a) Ischaemic stroke with known prior AF

	Premorbid $CHADS_2$										
	**0**	**1**	**2**	**3**	**4**	**5**	**6**	**Total**			
**Premorbid Warfarin use: Yes**	3	10	12	14	5	1	1	46			
**No**	15	37	83	55	29	8	1	228			
**Total**	18	47	95	69	34	9	2	274			
	**Premorbid CHA_2DS_2VASc score**										
	**0**	**1**	**2**	**3**	**4**	**5**	**6**	**7**	**8**	**9**	**Total**
**Premorbid Warfarin use: Yes**	1	2	5	9	12	9	5	2	0	1	46
**No**	4	10	13	44	68	47	28	11	3	0	228
**Total**	5	12	18	53	80	56	33	13	3	1	274
	**Premorbid HAS-BLED score**										
	**0**	**1**	**2**	**3**	**4**	**Total**					
**Premorbid Warfarin use: Yes**	3	25	14	3	1	46					
**No**	3	119	82	20	4	228					
**Total**	6	144	96	23	5	274					

S11b) PVE with known prior AF

	Premorbid $CHADS_2$							
	**0**	**1**	**2**	**3**	**4**	**5**	**6**	**Total**
**Premorbid Warfarin use: Yes**	1	2	3	3	0	1	0	10
**No**	1	10	11	13	12	4	1	52
**Total**	2	12	14	16	12	5	1	62

	Premorbid CHA_2DS_2VASc score									
	**0**	**1**	**2**	**3**	**4**	**5**	**6**	**7**	**8**	**Total**
**Premorbid Warfarin use: Yes**	0	0	1	2	2	3	1	1	0	10
**No**	1	0	4	4	10	10	13	7	3	52
**Total**	1	0	5	6	12	13	14	8	3	62

	Premorbid HAS-BLED score					
	**0**	**1**	**2**	**3**	**4**	**Total**
**Premorbid Warfarin use: Yes**	0	5	2	3	0	10
**No**	0	8	26	12	6	52
**Total**	0	13	28	15	6	62

Supplementary table S12: Comparison of premorbid $CHADS_2$ and HAS-BLED scores in patients with incident ischaemic stroke or peripheral embolic vascular event and known prior AF but not on anticoagulation in OXVASC.

(a) All ages

		Premorbid HAS-BLED score (/9)					Total
		0	1	2	3	4	
Premorbid $CHADS_2$ (/6)	0	2	7	6	1	0	16
	1	1	28	13	5	0	47
	2	0	43	40	9	2	94
	3	0	31	24	8	5	68
	4	0	15	16	8	2	41
	5	0	3	7	1	1	12
	6	0	0	2	0	0	2
Total		3	127	108	32	10	280

(b) Age ≥80

		Premorbid HAS-BLED score (/9)					Total
		0	1	2	3	4	
Premorbid CHADS2 (/6)	1	0	16	6	0	0	22
	2	0	34	26	7	2	69
	3	0	25	17	7	2	51
	4	0	12	14	7	1	34
	5	0	3	6	1	1	11
	6	0	0	2	0	0	2
Total		0	90	71	22	6	189

(c) All ages and excluding absolute or relative contraindication

		Premorbid HAS-BLED score (/9)					Total
		0	1	2	3	4	
Premorbid $CHADS_2$ (/6)	0	1	4	4	1	0	10
	1	1	18	5	3	0	27
	2	0	24	21	3	2	50
	3	0	21	15	8	3	47
	4	0	8	10	6	2	26
	5	0	3	5	1	1	10
Total		2	78	60	22	8	170

(d) Age ≥80 and excluding absolute or relative contraindication

		Premorbid HAS-BLED score (/9)					Total
		0	1	2	3	4	
Premorbid $CHADS_2$ (/6)	1	0	11	3	0	0	14
	2	0	20	11	3	2	36
	3	0	15	12	7	1	35
	4	0	6	9	5	1	21
	5	0	3	4	1	1	9
Total		0	55	39	16	5	115

Supplementary table S13: Reasons for no premorbid warfarin use in patients at all ages and at age ≥80 years with incident ischaemic stroke or peripheral embolic vascular event, known prior AF and $CHADS_2$ score ≥2 in OXVASC.

	Number (%)	
	All ages (n=217)	Age ≥80 (n=167)
No explanation	135 (62.2)	103 (61.7)
Patient refusal	21 (9.7)	15 (9.0)
Paroxysmal AF	7 (3.2)	5 (3.0)
In clinical trial	1 (0.5)	1 (0.6)
Previous cardioversion	1 (0.5)	0 (0)
Low $CHADS_2$ score	1 (0.5)	0 (0)
**Absolute contraindication**		
Multiple contraindications	4 (1.8)	3 (1.8)
Recent, active bleed	2 (0.9)	2 (1.2)
Non-compliant	1 (0.5)	1 (0.6)
**Relative contraindication**		
Previous bleed	14 (6.5)	12 (7.2)
Risk of falls	12 (5.5)	10 (6.0)
Dementia	7 (3.2)	7 (4.2)
Physician perception of unsuitability	4 (1.8)	2 (1.2)
Concurrent cancer	2 (0.9)	1 (0.6)
Anaemia	2 (0.9)	2 (1.2)
Renal or liver impairment	1 (0.5)	1 (0.6)
Old age	1 (0.5)	1 (0.6)
Frequent seizures	1 (0.5)	1 (0.6)

Supplementary table S14: Reasons for stopping previous warfarin at any point prior to an embolic event in OXVASC.

All ages

	Frequency	Percent
1. OAC never started	258	92.1
2. Stopped due to absolute contraindication	3	1.1
3. Stopped due to relative contraindication	7	2.5
4. Stopped for minor illness but forgot to restart	1	0.4
5. Stopped OAC for no apparent reason	5	1.8
6. Stopped or changed to antiplatelets as in sinus rhythm	2	0.7
7. Stopped as patient later declined	1	0.4
8. Changed to antiplatelet for no obvious reason	3	1.1
Total	280	100.0

Age ≥80

	Frequency	Percent
1. OAC never started	179	94.7
2. Stopped due to absolute contraindication	3	1.6
3. Stopped due to relative contraindication	2	1.1
4. Stopped for minor illness but forgot to restart	1	0.5
5. Stopped OAC for no apparent reason	1	0.5
6. Stopped or changed to antiplatelets as in sinus rhythm	1	0.5
7. Stopped as patient later declined	0	0
8. Changed to antiplatelet for no obvious reason	2	1.1
Total	189	100.0

Supplementary table S15a: Changes in disability status (modified Ranking scale – mRS) in patients with an incident ischaemic stroke or peripheral embolic vascular event and any AF but not on premorbid anticoagulants

All ages

All events		6 month mRS				
		0-2	3	4-5	6	Total
Pre-morbid mRS	0-2	112	32	34	80	258
	3	3	21	11	67	102
	4-5	0	0	10	27	37
Total		115	53	55	174	397

Ischaemic stroke		6 month mRS				Total
		0-2	3	4-5	6	0
Pre-morbid mRS	0-2	102	29	32	62	225
	3	3	21	7	49	80
	4-5	0	0	10	21	31
Total		105	50	49	132	336

Age ≥80

All events		6 month mRS				
		0-2	3	4-5	6	Total
Pre-morbid mRS	0-2	40	16	23	58	137
	3	2	16	9	56	83
	4-5	0	0	8	24	32
Total		42	32	40	138	252

Ischaemic stroke		6 month mRS				Total
		0-2	3	4-5	6	0
Pre-morbid mRS	0-2	38	14	21	47	120
	3	2	16	5	40	63
	4-5	0	0	8	18	26
Total		40	30	34	105	209

Supplementary table S15b: Changes in disability status (modified Ranking scale – mRS) in patients with an incident ischaemic stroke or peripheral embolic vascular event and known prior AF but not on premorbid anticoagulants

All ages

All events		6 month mRS				
		0-2	3	4-5	6	Total
Pre-morbid mRS	0-2	70	23	21	64	178
	3	2	12	8	54	76
	4-5	0	0	7	19	26
Total		72	35	36	137	280

Ischaemic stroke		6 month mRS				Total
		0-2	3	4-5	6	0
Pre-morbid mRS	0-2	62	21	19	49	151
	3	2	12	6	37	57
	4-5	0	0	7	13	20
Total		64	33	32	99	228

Age ≥80

All events		6 month mRS				
		0-2	3	4-5	6	Total
Pre-morbid mRS	0-2	26	12	14	47	99
	3	2	10	7	48	67
	4-5	0	0	5	18	23
Total		28	22	26	113	189

Ischaemic stroke		6 month mRS				Total
		0-2	3	4-5	6	0
Pre-morbid mRS						
	0-2	24	10	12	38	84
	3	2	10	5	32	49
	4-5	0	0	5	12	17
Total		26	20	22	82	150

Supplementary figure S1a-f: Premorbid warfarin use in OXVASC patients with known prior AF who had a fatal/disabling embolic event or were institutionalised at 6 months follow-up.

S1a: Premorbid warfarin use in OXVASC patients at age ≥80 with known prior AF who had a fatal/disabling embolic event or were institutionalised at 6 months follow-up. Total AF embolic events disabled/dead=230/449; Total AF embolic events institutionalized/dead=185/325

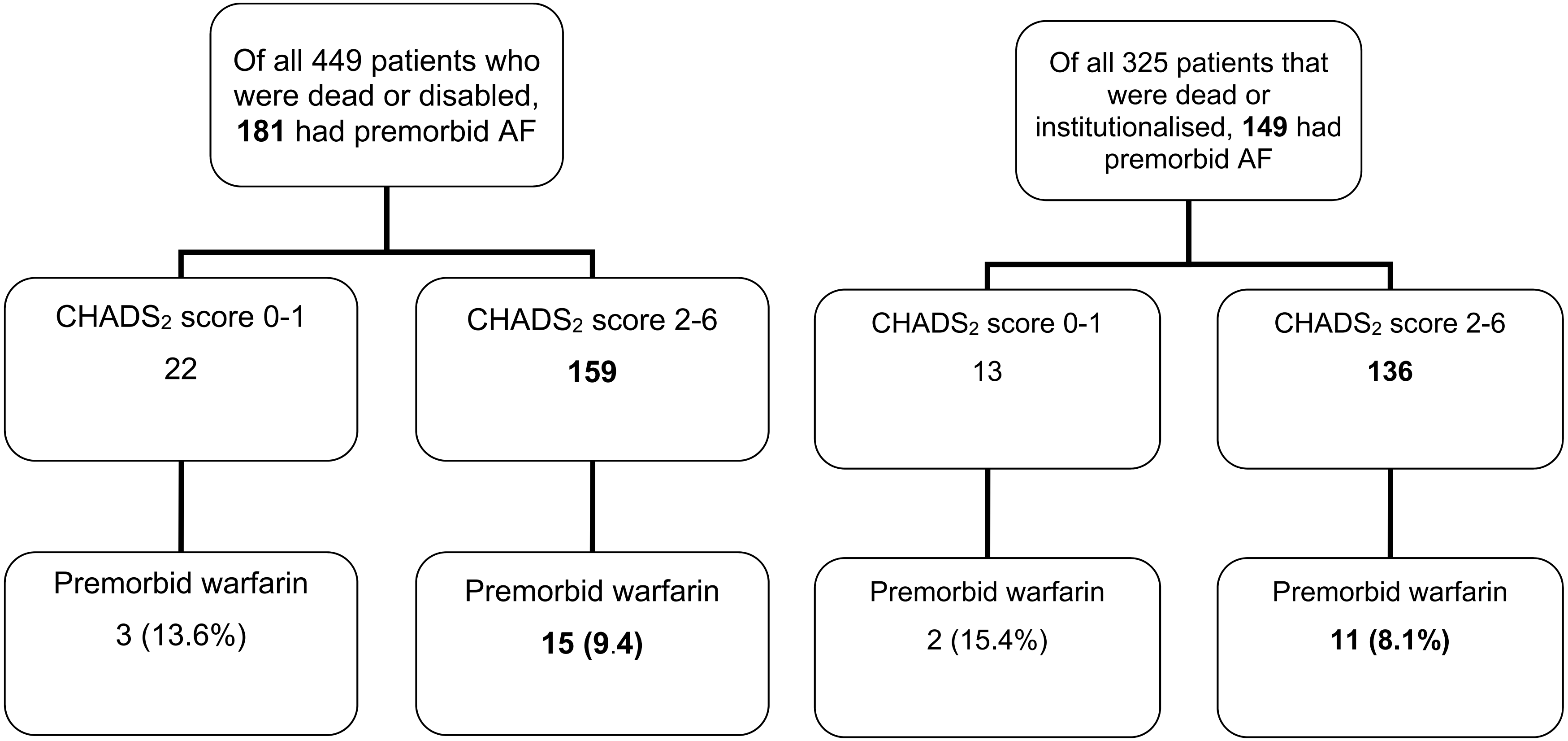

S1b: Premorbid warfarin use in OXVASC patients with known prior AF who had a fatal or disabling embolic event or were institutionalised at 6 months follow-up. Total AF embolic events disabled/dead=325/729; Total AF embolic events institutionalized/dead=240/471

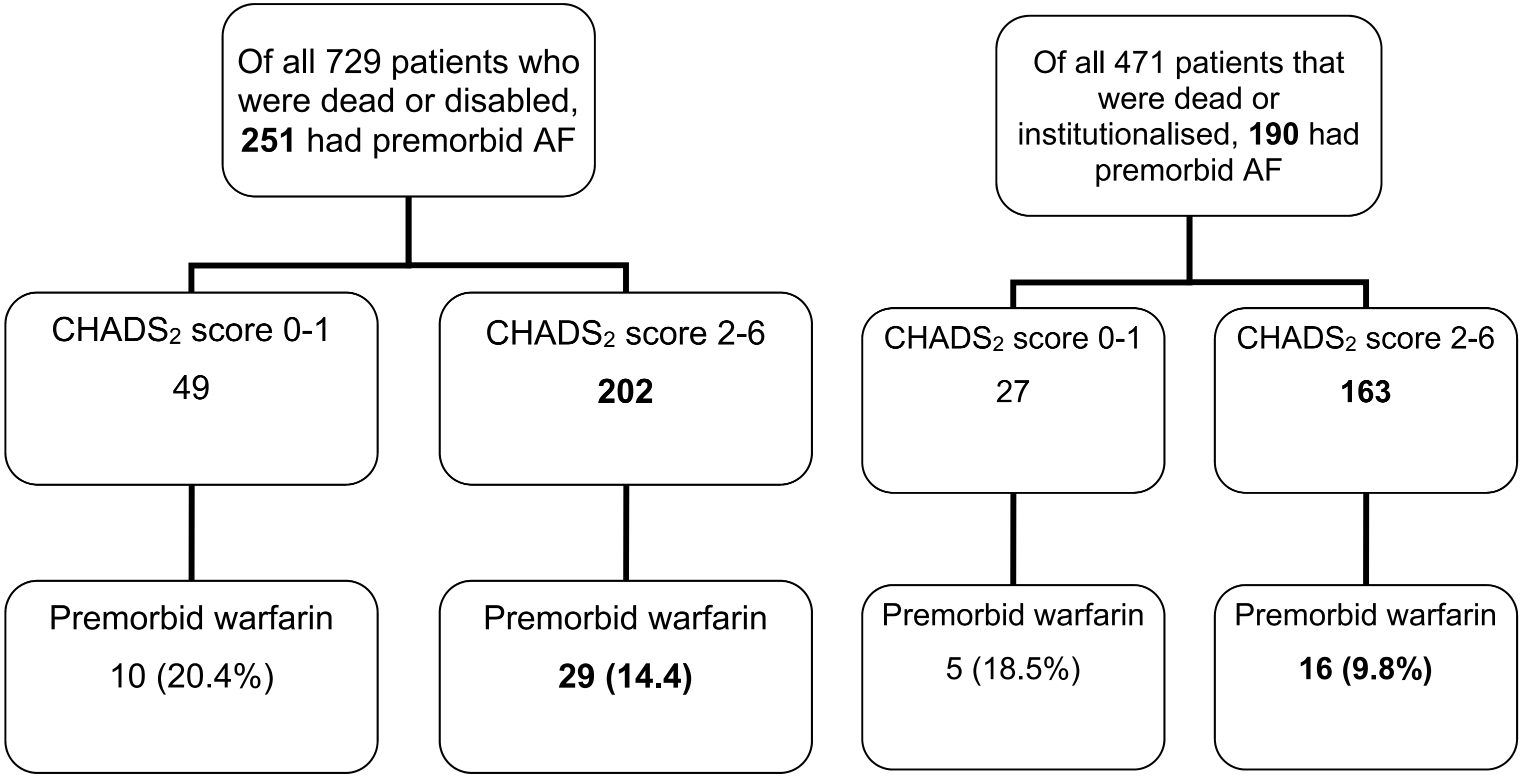

S1c: Premorbid warfarin use in OXVASC patients at age ≥80 with known prior AF who had a fatal/disabling ischaemic stroke or were institutionalised at 6 months follow-up. Total AFIIS (AF-related incident ischaemic stroke) disabled/dead =183/369; Total AFIIS institutionalized/dead =142/253

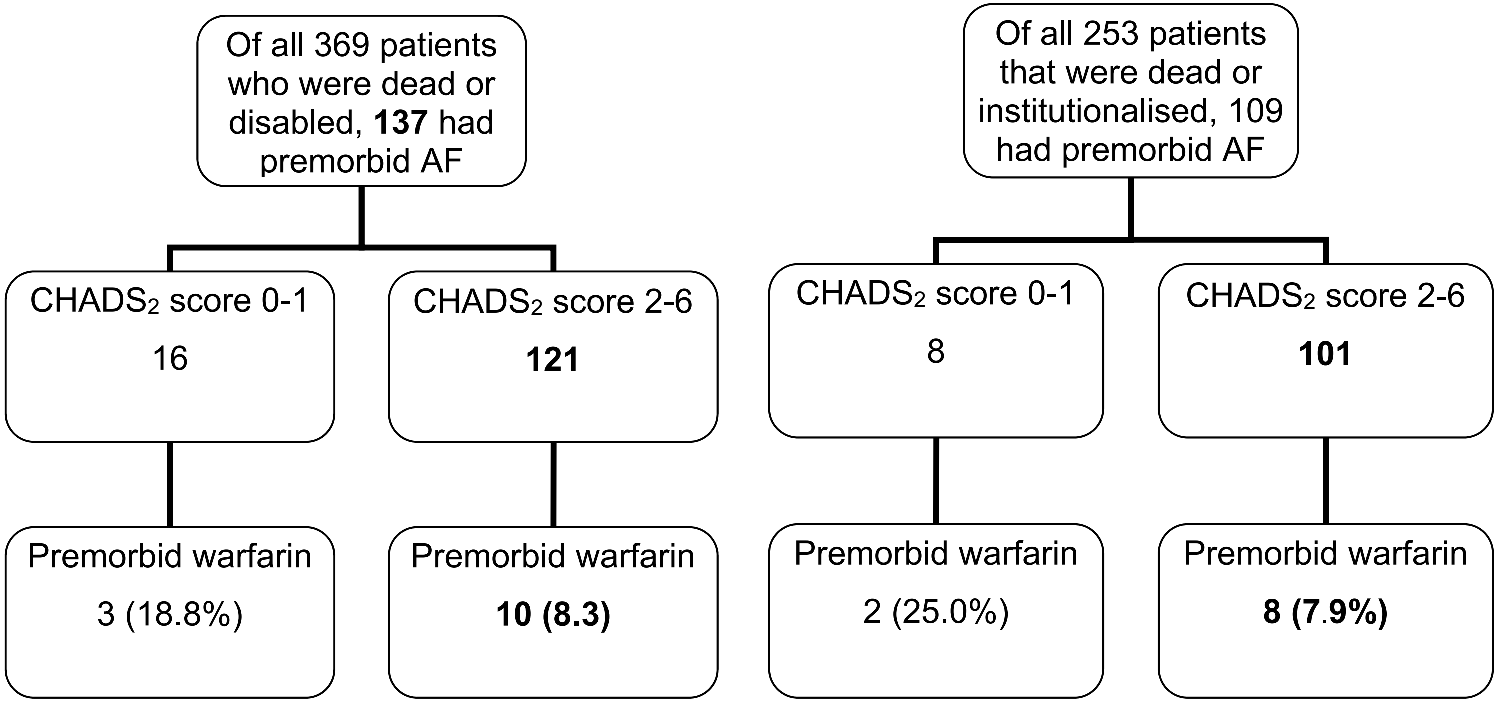

S1d: Premorbid warfarin use in OXVASC patients with known prior AF who had a fatal/disabling ischaemic stroke or were institutionalised at 6 months follow-up. Total AFIIS disabled/death=262; Total AFIIS institutionalised/dead=186

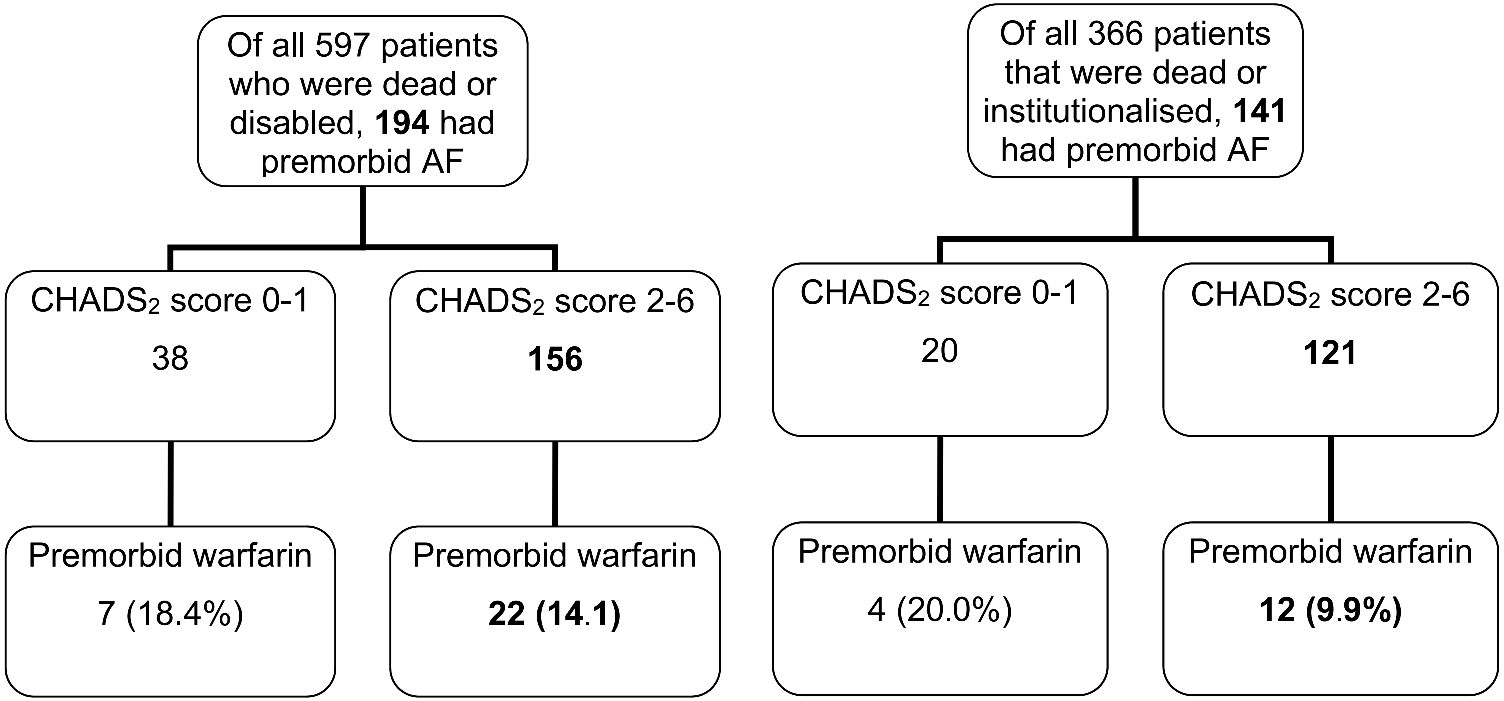

S1e: Premorbid warfarin use in OXVASC patients at age ≥80 with known prior AF who had a fatal/disabling (6M mRS=3-6) peripheral embolic vascular event or were institutionalised at 6 months follow-up.

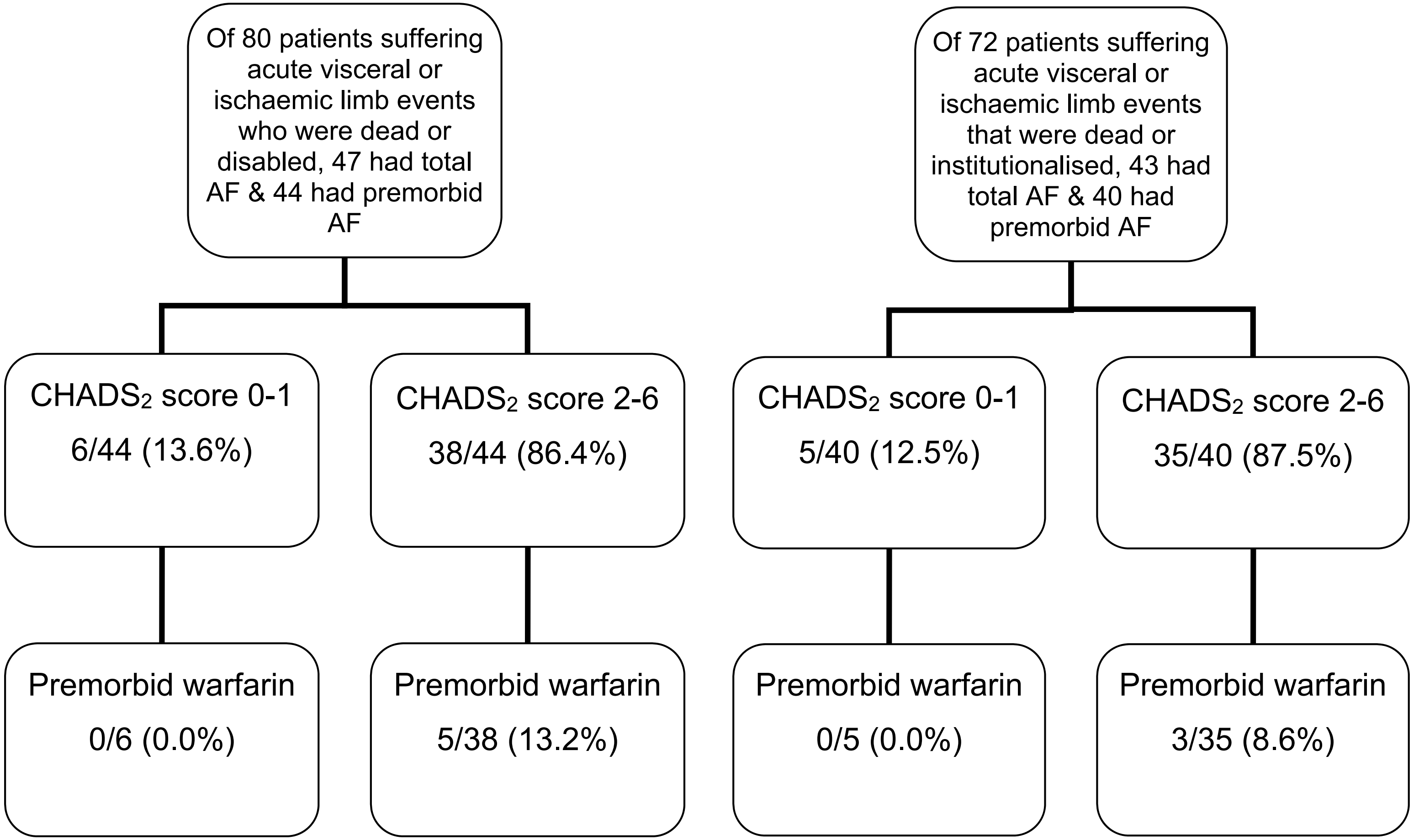

S1f: Premorbid warfarin use in OXVASC patients with known prior AF who had a fatal/disabling (6M mRS=3-6) peripheral embolic vascular event or were institutionalised at 6 months follow-up.

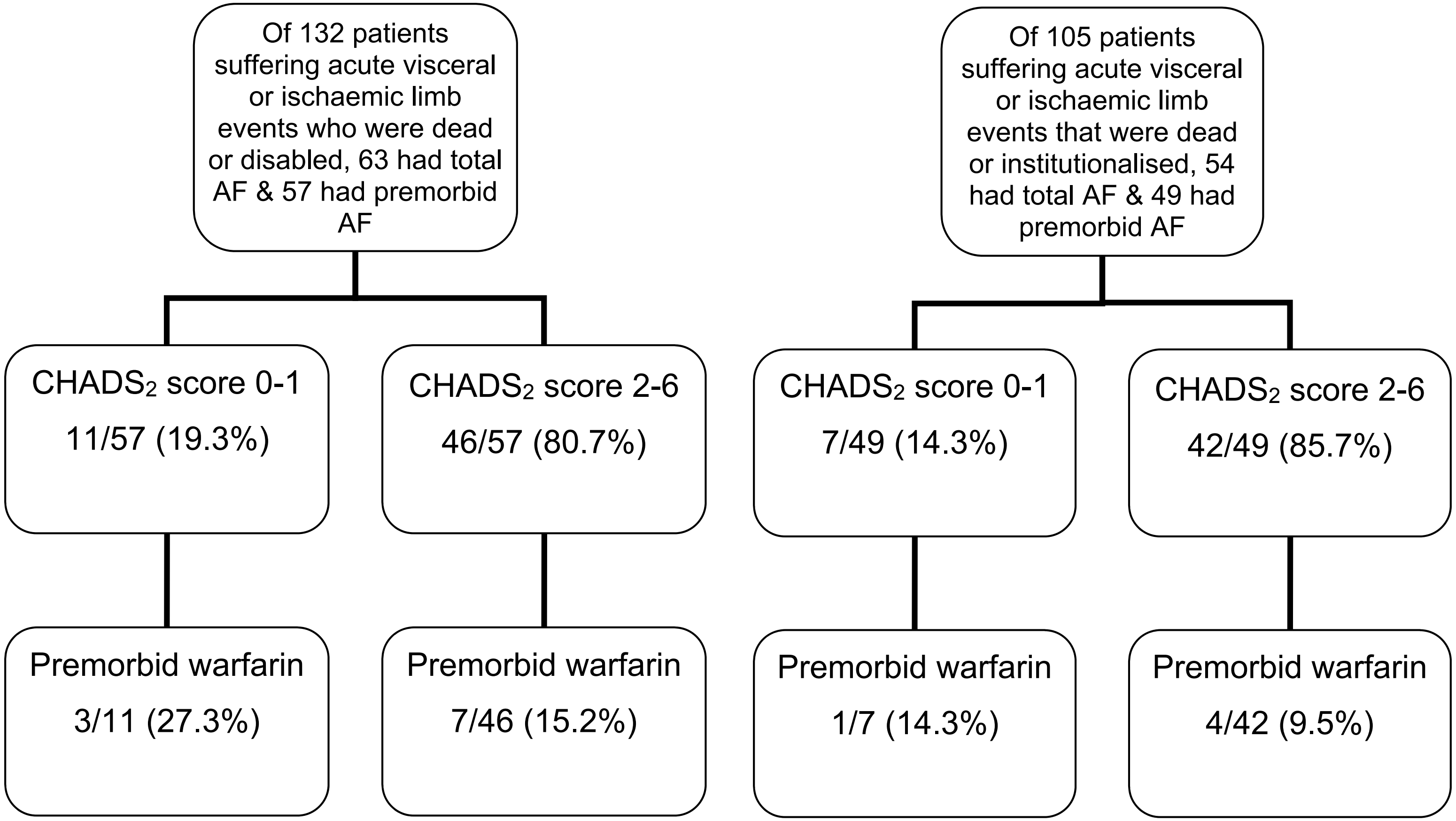

Supplementary figure S2: Rates of prior anticoagulation in patients with incident stroke in OXVASC and other recent stroke incidence studies; OAC=oral anticoagulation; *incident ischaemic stroke

Studies	OAC / Premorbid AF	% OAC	95% CI
2001-5 Barbados	19 / 64	29.7	18.5-40.9
2002-3 Auckland, NZ	71 / 289	24.6	19.6-29.5
2000-6 Dijon, France*	30 / 139	21.6	14.7-28.4
2005-6 North Dublin*	16 / 62	25.8	14.9-36.7
2006-7 Ludwigshafen, Germany*	42 / 121	34.7	26.2-43.2
2002-12 OXVASC, UK*	46 / 274	16.8	12.4-21.2
TOTAL	224 / 949	23.6	18.6-28.6
**Heterogeneity**	**p= 0.004476**		

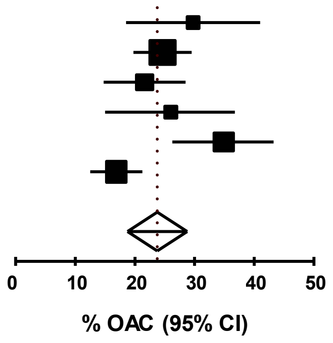

Supplement: Web supplement [file jnnp-2015-311947-s1.pdf]
